# Synthesis of zearalenone-16-β,D-glucoside and zearalenone-16-sulfate: A tale of protecting resorcylic acid lactones for regiocontrolled conjugation

**DOI:** 10.3762/bjoc.10.112

**Published:** 2014-05-15

**Authors:** Hannes Mikula, Julia Weber, Dennis Svatunek, Philipp Skrinjar, Gerhard Adam, Rudolf Krska, Christian Hametner, Johannes Fröhlich

**Affiliations:** 1 Institute of Applied Synthetic Chemistry, Vienna University of Technology (VUT), Getreidemarkt 9/163, A-1060 Vienna, Austria; 2 Department of Applied Genetics and Cell Biology, University of Natural Resources and Life Sciences, Vienna (BOKU), Konrad Lorenz Str. 24, 3430 Tulln, Austria; 3 Center for Analytical Chemistry, Department for Agrobiotechnology (IFA-Tulln), University of Natural Resources and Life Sciences, Vienna (BOKU), Konrad Lorenz Str. 20, 3430 Tulln, Austria

**Keywords:** glycosylation, masked mycotoxins, resorcylic acid esters, sulfation, zearalenone

## Abstract

The development of a reliable procedure for the synthesis of the 16-glucoside and 16-sulfate of the resorcylic acid lactone (RAL) type compound zearalenone is presented. Different protective group strategies were considered and applied to enable the preparation of glucosides and sulfates that are difficult to access up to now. Acetyl and *p*-methoxybenzyl protection led to undesired results and were shown to be inappropriate. Finally, triisopropylsilyl-protected zearalenone was successfully used as intermediate for the first synthesis of the corresponding mycotoxin glucoside and sulfate that are highly valuable as reference materials for further studies in the emerging field of masked mycotoxins. Furthermore, high stability was observed for aryl sulfates prepared as tetrabutylammonium salts. Overall, these findings should be applicable for the synthesis of similar RAL type and natural product conjugates.

## Introduction

**Figure 1:**
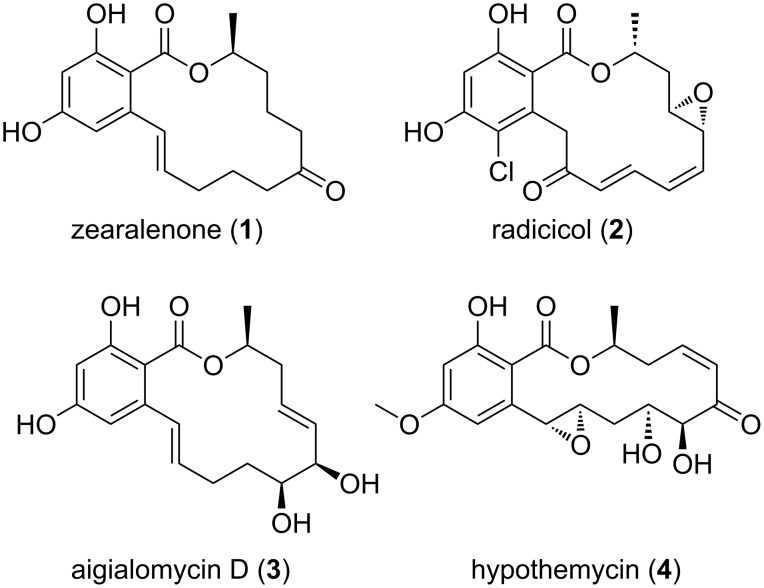
Structure of selected RAL type fungal secondary metabolites.

Resorcylic acid lactones (RALs, Figure 1), a compound class of benzannulated macrolides, are pharmacologically active secondary metabolites produced by a variety of different fungal species [1]. Zearalenone (ZEN, **1**) is a well-known RAL type mycotoxin for which maximum tolerated levels in food and feed were enacted and recommended, respectively, in Europe [2–3]. ZEN is produced by several plant pathogenic *Fusarium* species, including *Fusarium graminearum* and *Fusarium culmorum*. These species, which are the most frequently occurring toxin-producing fungi of the northern temperate zone, are commonly found in cereals and crops throughout the world [4–5]. Significant levels of ZEN are prevalently found in grains such as maize, wheat, and rice [6]. It is known that ZEN can cause problems of the reproductive tract (e.g., impaired fertility) in animals [7]. Physiological studies revealed binding of ZEN to recombinant human estrogen receptors [8] and have furthermore shown a ZEN-induced stimulation of the growth of human breast cancer cells [9].

**Figure 2:**
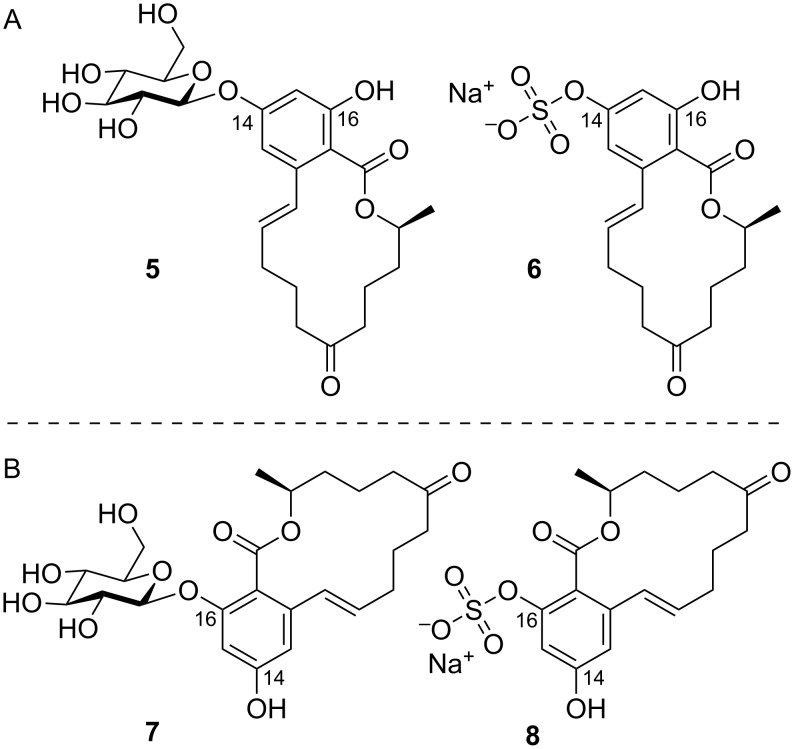
Structures of zearalenone conjugates: (A) ZEN-14-β,D-glucoside (**5**) and ZEN-14-sulfate (**6**), (B) ZEN-16-β,D-glucoside (**7**) and ZEN-16-sulfate (**8**). Sulfates shown as sodium salts.

Additionally, masked mycotoxins, especially altered derivatives formed through conjugation to sugar moieties or sulfate, emerge after metabolization by living plants. Due to changed chemical structures and properties compared to the parent mycotoxins, these conjugates can usually not be detected applying standard analytical techniques [10–11]. Responsible biochemical transformations are catalyzed usually by enzymes within detoxification processes [12]. Schneweis et al. reported the occurrence of ZEN-14-β,D-glucoside (**5**, Figure 2A) in wheat [13] and the first chemical synthesis of this compound applying phase transfer glycosylation has been reported by Grabley et al. [14]. ZEN-14-sulfate (**6**, Figure 2A) was first isolated from *F. graminearum*-inoculated rice [15] and both, the glucoside **5** and the sulfate **6**, were identified as ZEN metabolites in *Arabidopsis thaliana* [16]. These conjugates are easily hydrolyzed back to the parental mycotoxin during digestion of contaminated grain, and should therefore be considered as masked mycotoxins [12]. Recently it has been shown that ZEN treated barley, wheat and *Brachypodium distachyon* cells produce both the ZEN-14 and the ZEN-16-glucoside, with up to 18-fold higher levels of ZEN-16-glucoside than ZEN-14-glucoside in barley roots [17]. We therefore intended to develop a synthetic method for regiocontrolled conjugation of ZEN. Basically, the RAL type moiety of ZEN contains two possible sites for glycosylation/sulfation, but due to the higher reactivity of the phenol group at position 14, reactions at this site are strongly favored compared to conjugation at C16–OH [18–20]. Although natural products containing a RAL type moiety conjugated at the phenol group in ortho position to the carboxyl group (2’-OH) were already detected and identified [21–25], to the best of our knowledge there are no reliable synthetic procedures and strategies towards this class of compounds described in the literature so far. The synthesis of the natural glucoside delphoside by Saeed was performed using methyl ether protection at O-6 of the isocoumarin core structure during glucosylation and rather harsh unfavorable demethylation with boron tribromide in the last step [26]. Without structure verification and characterization ZEN-16-β,D-glucoside (**7**, Figure 2B) was tentatively identified as a byproduct of the Königs–Knorr glucosylation of ZEN for preparation of ZEN-14-glucoside [27]. In the course of ongoing research in the emerging field of masked mycotoxins, we were able to prepare ZEN-16-β,D-glucoside (**7**) and ZEN-16-sulfate (**8**, Figure 2B) in reasonable amounts after the development of reliable procedures that should be generally applicable to resorcylic acid lactones. Additionally the first chemical synthesis of the ZEN-derivative 14-O-acetylzearalenone (14-AcZEN, produced by some Fusarium strains) [28] is reported.

## Results and Discussion

**Scheme 1:**
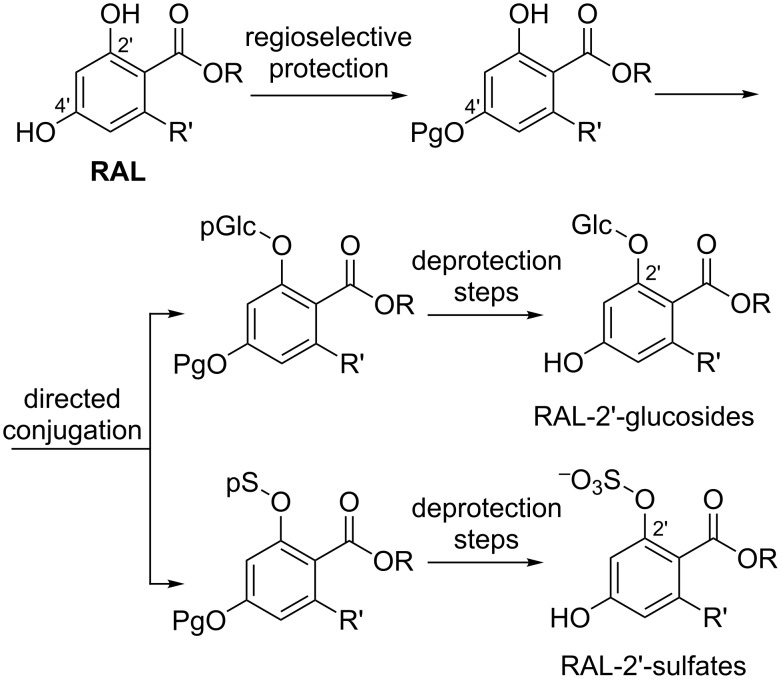
General strategy for the synthesis of RAL-2’-conjugation (Pg: protective group, pGlc: protected glucose moiety, pS: protected sulfate, Glc: β,D-glucoside).

The general strategy for regiocontrolled conjugation at position 2’ of resorcylic acid esters and lactones is shown in Scheme 1. Regioselective protection of the more nucleophilic 4’-phenol and subsequent glucosylation or sulfation should lead to the desired products.

**Scheme 2:**
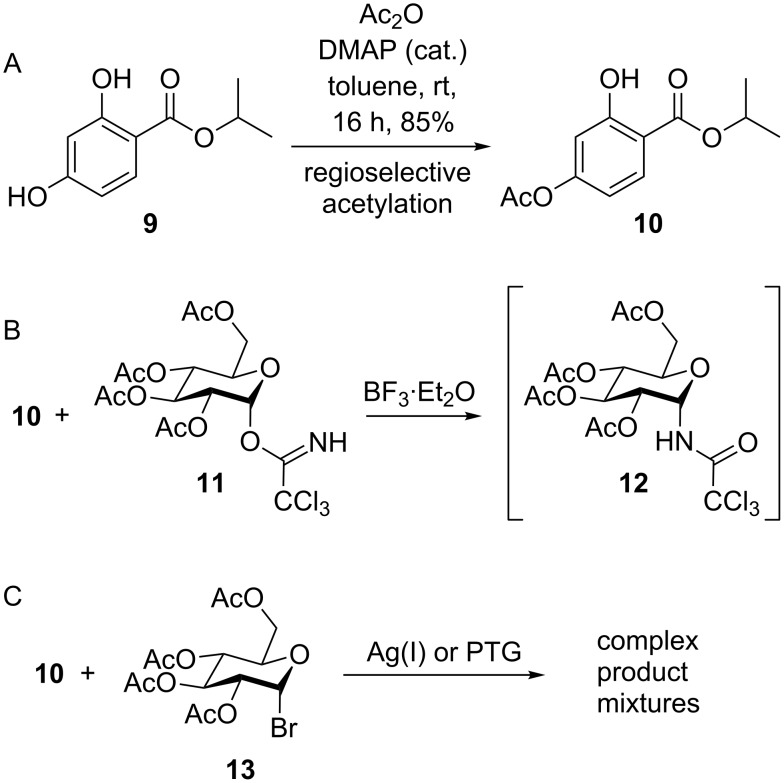
(A) Regioselective acetylation of resorcylic acid ester **9**. (B) Lewis acid mediated glucosylation; no conversion of **10**, partially rearrangement of **11** to glucosyl acetamide **12**. (C) Königs–Knorr glucosylation; Ag(I): Ag_2_CO_3_ or Ag_2_O, CH_2_Cl_2_ or MeCN; PTG: phase transfer glucosylation, NaOH, tetrabutylammonium bromide, pH 10–11, CHCl_3_/H_2_O.

For the development of a reliable protective group strategy and subsequent reaction optimization, 2,4-dihydroxybenzoic acid isopropyl ester (**9**) [29] was used as a RAL mimic. For protection of the 4-OH group we first considered an acetyl group that could be regioselectively introduced by reaction of **9** with acetic anhydride and catalytic amounts of 4-(dimethylamino)pyridine (DMAP) to obtain the acetylated RAL mimic **10** (Scheme 2A). Different methods for glycosylation were investigated using acetyl-protected glucosyl donors since diastereoselective β-conjugation, which is needed for the preparation of glucosides formed during phase II metabolism, is commonly achieved applying the participation of acyl groups at O-2 of the glycosyl donor (anchimeric effect) [30]. Lewis acid-mediated glucosylation using the trichloroacetimidate donor **11** according to the procedure of Saeed [26] did not lead to the desired product, which can be explained by the weak nucleophilicity of the 2-OH group of the acceptor **10**. This assumption was supported by detection of the glucosyl acetamide **12**, which is known to be formed by rearrangement of **11** when activated in the presence of a weak acceptor (Scheme 2B) [31–34]. Königs–Knorr glucosylation, which in general is most frequently used for the glucosylation of phenols, using commercially available bromo sugar **13** activated by silver(I) salts or under phase transfer conditions led to complex product mixtures (Scheme 2C).

**Scheme 3:**
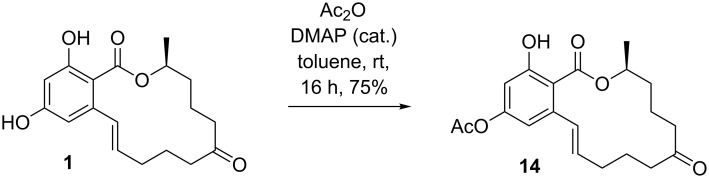
Regioselective acetylation of ZEN (**1**) affording 14-O-acetylzearalenone (**14**).

Nevertheless, the procedure for selective acetylation of resorcylic acid esters and lactones was applied for the first synthesis of 14-O-acetylzearalenone (**14**) (Scheme 3).

**Scheme 4:**
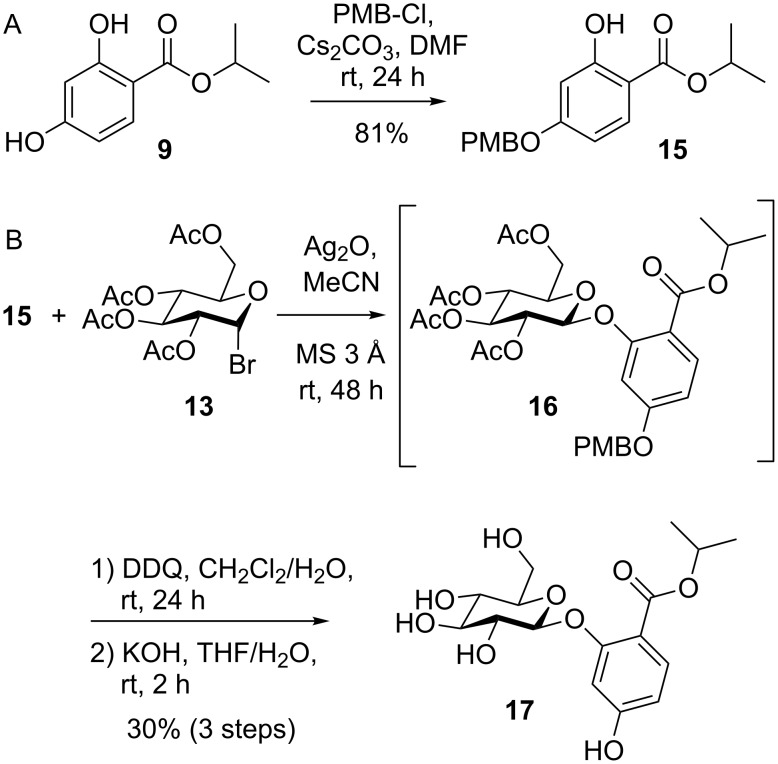
(A) Regioselective p-methoxybenzylation of **9**. (B) Synthesis of the ZEN-16-Glc mimic **17**.

To avoid undesired cleavage of the acetyl group during glucosylation of **10**, *p*-methoxybenzyl (PMB) protection was applied instead, since the PMB group was considered to be inert to the reactions conditions of the Königs–Knorr procedure, thus forcing conjugation at position 2. Regioselective *p*-methoxybenzylation of **9** was achieved by reaction with PMB-Cl and Cs_2_CO_3_ in dry DMF after optimization in terms of base and solvent type (Scheme 4A). Königs–Knorr glucosylation of the PMB-protected mimic **15** afforded **16**, which was deprotected using 2,3-dichloro-5,6-dicyano-*p*-benzoquinone (DDQ) for oxidative PMB cleavage and subsequent ester saponification to yield the desired glucoside **17** in an overall yield of 30% (Scheme 4B).

**Scheme 5:**
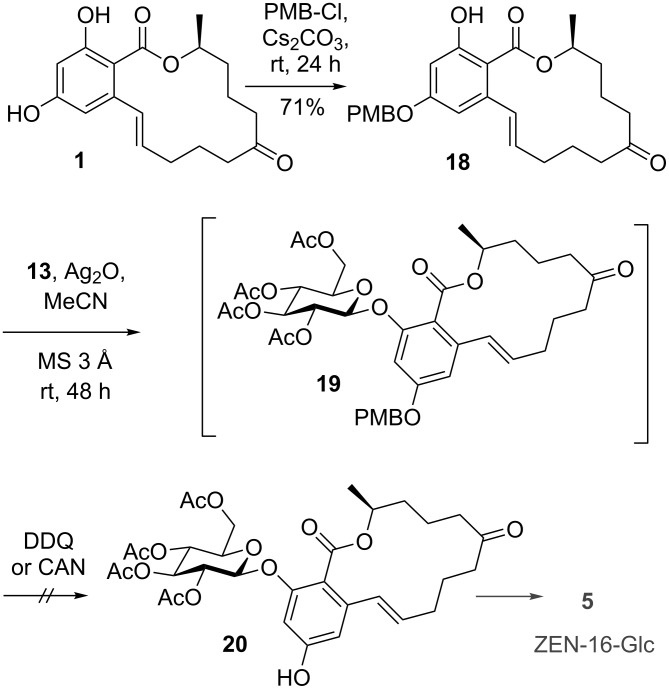
Regioselective protection of ZEN (**1**) and failed PMB cleavage of the glucosylated intermediate **19**.

Applying this procedure to ZEN (**1**) afforded the glucosylated intermediate **19** after Königs–Knorr glucosylation of 14-PMB-ZEN (**18**), but subsequent deprotection using DDQ did not afford the desired product. Also an alternative procedure for oxidative PMB cleavage using cerium ammonium nitrate (CAN) did not lead to the formation of the deprotected compound **20** (Scheme 5). Beside unreacted **19**, LC–MS/MS analysis showed the formation of a product with an *m*/*z* value 16 amu higher than calculated for **19** indicating oxidation of this intermediate but no deprotection.

Since the DDQ-promoted cleavage of phenolic PMB ethers can be complicated by overoxidation, especially with electron-rich phenolic compounds [35], we assume a significant effect of the conjugated olefinic double bond at C-6’ of the resorcylic acid moiety being responsible for the observed different behavior of ZEN (**1**) and the ZEN mimic **9**.

**Scheme 6:**
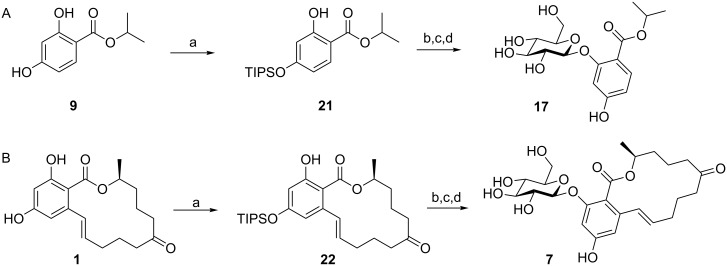
Synthesis of (A) ZEN mimic glucoside **17** and (B) ZEN-16-β,D-glucoside (**7**); a: TIPS-Cl, imidazole, 16 h, rt, >99% for **21** and **22**; b: **13**, Ag_2_O, MeCN, 96 h, rt; c: TBAF, AcOH, THF, 24 h; d: KOH, THF/H_2_O, 4 h, rt, 41% for **17**, 34% for **7** (3 steps).

After this second setback the protective group strategy was changed again within a third approach. Considering steric hindrance and methods for selective deprotection under relatively mild conditions led us to the use of triisopropylsilyl (TIPS) protection for regiocontrolled glucosylation of resorcylic acid esters and lactones. Regioselective silylation of **9** and ZEN (**1**) was readily achieved affording compounds **21** and **22** (14-TIPS-ZEN), respectively, in nearly quantitative yields by reaction with TIPS-Cl and imidazole in dry CH_2_Cl_2_. Applying this strategy we were finally able to accomplish the synthesis of the ZEN mimic glucoside **17** (Scheme 6A) as well as of the target compound ZEN-16-β,D-glucoside (**7**) as shown in Scheme 6B. Reasonable yields of 41% (**17**) and 34% (**7**), respectively, were obtained applying an optimized purification protocol.

**Scheme 7:**
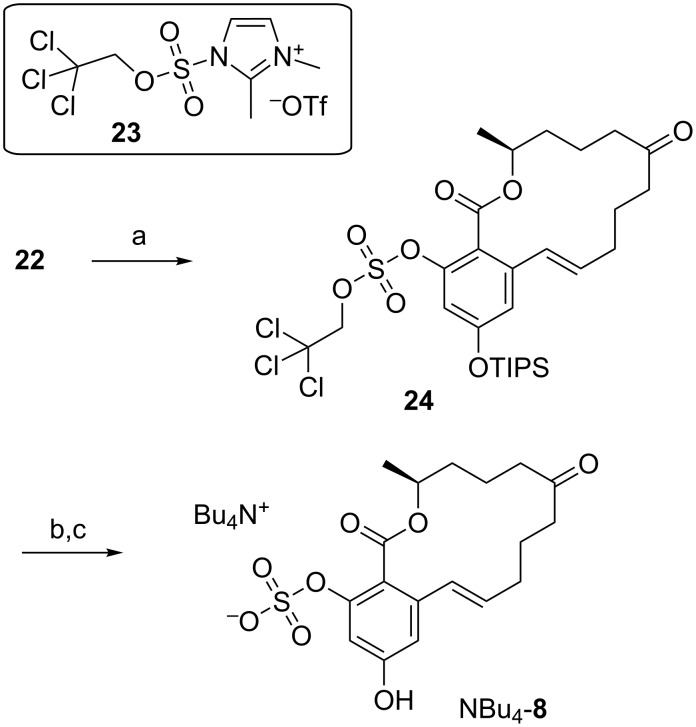
Chemical sulfation using the 2,2,2-trichloroethyl (TCE)-protected sulfuryl imidazolium salt **23** yielding ZEN-16-sulfate (**8**) as tetrabutylammonium salt; a: **23**, 1,2-dimethylimidazole, CH_2_Cl_2_, 12 h, rt, 91%, b: TBAF, AcOH, THF, 3 h, −10 °C, c: Zn, HCOONH_4_, MeOH, 16 h, rt, 65% (2 steps).

Additionally, TIPS protection was applied for the synthesis of ZEN-16-sulfate (**8**) using a procedure that was successfully applied for the synthesis of ZEN-14-sulfate (**6**) as described recently [20]. Reaction of **22** with the 2,2,2-trichloroethyl (TCE) protected sulfuryl imidazolium salt **23** [36–37] gave the protected sulfate **24**. TIPS cleavage and subsequent TCE deprotection using Zn/ammonium formate (HCOONH_4_) yielded the desired product. Interestingly, when using the crude intermediate after TIPS deprotection without purification directly in the second step, we obtained the tetrabutylammonium salt of ZEN-16-sulfate (NBu_4_-**8**) in good yield (65% over 2 steps) as shown in Scheme 7. The stability of this compound was significantly increased compared to the corresponding sodium salt, which is of great importance in terms of preparation of reference material and long term stability of appropriate standard solutions for further investigations.

## Conclusion

In summary, different methods for the regioselective protection of resorcylic acid esters and lactones were investigated for subsequent regiocontrolled glucosylation and sulfation. Whereas acetyl and *p*-methoxybenzyl protection led to undesired products, TIPS-protected RALs were successfully used as intermediates for the preparation of corresponding glucosides and sulfates applying the Königs–Knorr glucosylation and chemical sulfation using TCE-protected sulfuryl imidazolium salt **23**, respectively. These methods were used for the first chemical synthesis of the ZEN-16-conjugates **7** and **8** in reasonable amounts for ongoing research and further investigations in the field of conjugated/masked mycotoxins.

## Supporting Information

File 1 Experimental details (including remarks and general procedures), characterization data, copies of NMR spectra of new compounds, 2D NMR spectra of glucoside **7**.
