# Supplementary material for: Synthesis of zearalenone-16-β,D-glucoside and zearalenone-16-sulfate: A tale of protecting resorcylic acid lactones for regiocontrolled conjugation
Source: Beilstein J Org Chem. 2014 May 15;10:1129–34. doi: 10.3762/bjoc.10.112 (PMC4077526; doi:10.3762/bjoc.10.112)

Supporting Information

for

Synthesis of zearalenone-16-β,D-glucoside and zearalenone-16-sulfate: A tale of protecting resorcylic acid lactones for regiocontrolled conjugation

Hannes Mikula*[1], Julia Weber[1], Dennis Svatunek[1], Philipp Skrinjar[1], Gerhard Adam[2], Rudolf Krska[3], Christian Hametner[1] and Johannes Fröhlich[1]

Address: [1]Institute of Applied Synthetic Chemistry, Vienna University of Technology (VUT), Getreidemarkt 9/163, A-1060 Vienna, Austria, [2]Department of Applied Genetics and Cell Biology, University of Natural Resources and Life Sciences, Vienna (BOKU), Konrad Lorenz Str. 24, 3430 Tulln, Austria and [3]Center for Analytical Chemistry, Department for Agrobiotechnology (IFA-Tulln), University of Natural Resources and Life Sciences, Vienna (BOKU), Konrad Lorenz Str. 20, 3430 Tulln, Austria

* Corresponding author

Email: Hannes Mikula* - hannes.mikula@tuwien.ac.at

Experimental details (including remarks and general procedures), characterization data, copies of NMR spectra of new compounds, 2D NMR spectra of glucoside 7.

Content

1. **Experimental** S3

General remarks S3

General procedure A: regioselective acetylation of RAL type compounds S3

4-Acetoxy-2-hydroxybenzoic acid isopropyl ester (**10**) S4

14-O-Acetylzearalenone (**14**) S4

General procedure B: regioselective *p*-methoxybenzylation of RAL type compounds S4

2-Hydroxy-4-((*p*-methoxybenzyl)oxy)benzoic acid isopropyl ester (**15**) S5

14-O-(*p*-Methoxybenzyl)zearalenone (**18**) S5

General procedure C: regioselective TIPS-protection of RAL type compounds S6
2-Hydroxy-4-(triisopropylsilyloxy)benzoic acid isopropyl ester (**21**) S6
14-O-(Triisopropylsilyl)zearalenone (**22**) S6
General procedure D: Königs–Knorr glucosylation S7
5-Hydroxy-2-(isopropoxycarbonyl)phenyl-β,D-glucoside (**17**) S7
Zearalenone-16-β,D-glucoside (**7**) S8
16-O-((2,2,2,-Trichloroethoxy)sulfonyl)-14-O-(triisopropylsilyl)zearalenone (**24**) S8
Zearalenone-16-sulfate, tetrabutylammonium salt (NBu_4-**8**) S9

2. NMR spectra S11
Figure S1: COSY spectrum of **7** S11
Figure S2: HSQC spectrum of **7** S12
Figure S3: HMBC spectrum of **7** S12
Figure S4: ^{1}H NMR spectrum of **8** S13
Figure S5: ^{13}C NMR spectrum of **8** S13
Figure S6: ^{1}H NMR spectrum of **10** S14
Figure S7: ^{13}C NMR spectrum of **10** S14
Figure S8: ^{1}H NMR spectrum of **14** S15
Figure S9: ^{13}C NMR spectrum of **14** S15
Figure S10: ^{1}H NMR spectrum of **15** S16
Figure S11: ^{13}C NMR spectrum of **15** S16
Figure S12: ^{1}H NMR spectrum of **17** S17
Figure S13: ^{13}C NMR spectrum of **17** S17
Figure S14: ^{1}H NMR spectrum **of 18** S18
Figure S15: ^{13}C NMR spectrum of **18** S18
Figure S16: ^{1}H NMR spectrum of **21** S19
Figure S17: ^{13}C NMR spectrum of **21** S19
Figure S18: ^{1}H NMR spectrum of **22** S20
Figure S19: ^{13}C NMR spectrum of **22** S20
Figure S20: ^{1}H NMR spectrum of **24** S21
Figure S21: ^{13}C NMR spectrum of **24** S21

1. Experimental

General remarks

All reactions were performed under an argon atmosphere. The progress of reactions was monitored by thin-layer chromatography (TLC) over silica gel 60 F254 (Merck). The chromatograms were visualized by irradiation with ultraviolet light or by heat staining with ceric ammonium molybdate in ethanol/sulfuric acid. LC–ESI–MS/MS was performed on an HCT ion trap mass spectrometer (Bruker, Germany) in full scan mode. Chromatographic separation was done on a 1200 series HPLC system (Agilent Technologies, Germany) using a Luna RP-C18 column (3.0 × 150 mm, 3 µm particle size, Phenomenex, Germany) and application of pure substances was achieved using a TLC–MS interface (Camag, Germany). Preparative column chromatography was performed on silica gel 60 (Merck, 40-63 µm) or RP-C18 silica gel (Merck, 40–63 µm) using a Büchi Sepacore™ Flash System. NMR spectra were recorded on a Bruker DPX-200 MHz or Avance DRX-400 MHz spectrometer. Data were recorded and evaluated using TOPSPIN 1.3 (Bruker Biospin). All chemical shifts are given in ppm relative to tetramethylsilane. The calibration was done using residual solvent signals. Multiplicities are abbreviated as s (singlet), d (doublet), t (triplet), q (quartet), b (broad signal). Zearalenone was obtained from Fermentek (Israel) and all other chemicals were purchased from ABCR (Germany) or Sigma-Aldrich (Austria/Germany).

General procedure A: regioselective acetylation of RAL type compounds

To a solution of the RAL type compound (1.0 mmol) and DMAP (1.2 mg, 10 µmol) in dry toluene (5 mL) at 0 °C was slowly added a solution of Ac_2O (110 mg, 1.1 mmol) in dry toluene (1 mL). The reaction mixture was gradually warmed to room temperature and stirred for further 16 h. The solvent was removed under reduced pressure and the residue was purified by column chromatography (hexanes/EtOAc gradient elution) to yield the desired 4'-O-acetylated product.

4-Acetoxy-2-hydroxybenzoic acid isopropyl ester (10)

Following general procedure A, **10** was obtained as a white solid (203 mg, 85%); R_f 0.4 (hexanes/EtOAc, 9/1); ^{1}H NMR (200 MHz, $CDCl_3$) δ 11.07 (s, 1H, 2-OH), 7.84 (d, J = 8.8 Hz, 1H, H-6), 6.72 (d, J = 2.3 Hz, 1H, H-3), 6.62 (dd, J = 8.8, 2.3 Hz, 1H, H-5), 5.26 (sept., J = 6.2 Hz, 1H, COOCH), 2.27 (s, 3H, CH_3COO), 1.36 (d, J = 6.2 Hz, 6H, $(CH_3)_2CH$); ^{13}C NMR (50 MHz, $CDCl_3$) δ 169.2 (s), 168.5 (s), 162.9 (s), 156.1 (s), 131.0 (d), 112.9 (d), 110.7 (s), 110.4 (d), 69.3 (d), 21.8 (q), 21.1 (q); HRMS *m/z* calcd for $C_{12}H_{14}NaO_5^+$ $[M+Na]^+$ 261.0733, found 261.0750.

14-*O*-Acetylzearalenone (14)

Following general procedure A (reduced scale), **14** was obtained starting from ZEN (**1**, 32 mg, 0.1 mmol) as a white solid (27 mg, 75%); R_f 0.4 (hexanes/EtOAc, 2/1); ^{1}H NMR (200 MHz, $CDCl_3$) δ 11.88 (s, 1H, 16-OH), 7.00 (d, J = 15.6 Hz, 1H, H-12), 6.67-6.62 (m, 2H, H-13, H-15), 5.71 (ddd, J = 15.4, 10.1, 4.3 Hz, 1H, H-11), 5.11-4.93 (m, 1H, H-3), 2.81 (ddd, J = 18.7, 12.1, 2.5 Hz, 1H, H_a-8), 2.59 (m, 1H, H_a-6), 2.37-2.05 (m, 5H, H_a-5, H_b-6, H_b-8, H_{ab}-10), 2.29 (s, 3H, CH_3COO), 1.85-1.54 (m, 5H, H_{ab}-4, H_b-5, H_{ab}-9), 1.39 (d, J = 6.1 Hz, 3H, CH_3); ^{13}C NMR (50 MHz, $CDCl_3$) δ 211.0 (s, C-7), 171.1 (s, $CH_3\mathit{C}OO$), 168.8 (s, C-1), 164.6 (s, C-14), 154.9 (s, C-16), 143.4 (s, C-12a), 133.5 (d, C-11), 132.6 (d, C-12), 113.7 (d, C-13), 109.6 (d, C-15), 108.3 (s, C-16a), 74.1 (d, C-3), 43.0 (t, C-6), 36.7 (t, C-8), 34.7 (t, C-4), 31.1 (t, C-10), 22.2 (t, C-5), 21.2 (q, $\mathit{C}H_3COO$), 21.0 (t, C-9), 20.8 (q, CH_3); NMR data matched that reported previously by Munoz et al.[1] HRMS *m/z* calcd for $C_{20}H_{24}NaO_6^+$ $[M+Na]^+$ 383.1465, found 383.1466.

General procedure B: regioselective *p*-methoxybenzylation of RAL type compounds

To a solution of the RAL type compound (1 mmol) and Cs_2CO_3 (433 mg, 1.5 mmol) in dry DMF (5 mL) was slowly added a solution of *p*-methoxybenzyl chloride (PMB-Cl, 172 mg, 1.1 mmol) in dry DMF (1 mL). The reaction mixture was stirred at room temperature for 24 h, diluted with water (20 mL) and extracted with EtOAc (3 × 15 mL). The combined organic layer was washed with brine,

[1] Munoz, L.; Castro, J.L.; Cardelle, M.; Castedo, L.; Riguera, R. *Phytochemistry* **1989**, *28*, 83-85.

dried over Na_2SO_4 and concentrated. Column chromatography (hexane/EtOAc gradient elution) afforded the desired 4'-O-PMB protected compound.

2-Hydroxy-4-((*p*-methoxybenzyl)oxy)benzoic acid isopropyl ester (15)

Following general procedure B, **15** was obtained as a white solid (256 mg, 81%); R_f 0.8 (hexanes/EtOAc, 3/1); ^{1}H NMR (200 MHz, $CDCl_3$) δ 11.07 (s, 1H, 2-OH), 7.66 (d, J = 8.5 Hz, 1H, H-6), 7.26 (d, J = 8.5 Hz, 2H, PMB), 6.84 (d, J = 8.5 Hz, 2H, PMB), 6.46-6.36 (m, 2H, H-3, H-5), 5.17 (sept., J = 6.5 Hz, 1H, COOCH), 4.92 (s, 2H, OCH_2), 3.74 (s, 3H, OCH_3), 1.29 (d, J = 6.4 Hz, 6H, $(CH_3)_2CH$); ^{13}C NMR (50 MHz, $CDCl_3$) δ 169.6 (s), 164.6 (s), 163.8 (s), 159.6 (s), 131.2 (d), 129.3 (d), 128.1 (s), 114.1 (d), 108.0 (d), 106.1 (s), 101.6 (d), 69.9 (t), 68.7 (q), 55.3 (q), 21.9 (q); HRMS *m/z* calcd for $C_{18}H_{20}NaO_5^+$ $[M+Na]^+$ 339.1203, found 339.1195.

14-*O*-(*p*-Methoxybenzyl)zearalenone (18)

Following general procedure B (reduced scale), **18** was obtained starting from ZEN (**1**, 32 mg, 0.1 mmol) as a white solid (31 mg, 71%); R_f 0.6 (hexanes/EtOAc, 2/1); ^{1}H NMR (200 MHz, $CDCl_3$) δ 12.01 (s, 1H, 16-OH), 7.27 (d, J = 8.6 Hz, 2H, PMB), 6.94 (d, J = 14.9 Hz, 1H, H-12), 6.84 (d, J = 8.8 Hz, 2H, PMB), 6.46 (d, J = 2.6 Hz, 1H, H-13), 6.39 (d, J = 2.6 Hz, 1H, H-15), 5.60 (ddd, J = 15.5, 10.3, 4.0 Hz, 1H, H-11), 5.03-4.83 (m, 3H, H-3, OCH_2), 3.74 (s, 3H, OCH_3), 2.77 (ddd, J = 18.2, 12.2, 2.9 Hz, 1H, H_a-8), 2.60-2.45 (m, 1H, H_a-6), 2.37-1.96 (m, 5H, H_a-5, H_b-6, H_b-8, H_{ab}-10), 1.78-1.45 (m, 5H, H_{ab}-4, H_b-5, H_{ab}-9), 1.31 (d, J = 6.1 Hz, 3H, CH_3); ^{13}C NMR (50 MHz, $CDCl_3$) δ 211.0 (s, C-7), 171.4 (s, C-1), 165.6 (s, C-14), 163.2 (s, C-16), 159.6 (s, Ar-OCH_3), 143.3 (s, C-12a), 133.3 (d, C-11), 132.4 (d, C-12), 129.3 (d, PMB), 128.1 (s, PMB), 114.0 (d, PMB), 108.9 (d, C-13), 103.7 (s, C-16a), 100.8 (d, C-15), 73.4 (d, C-3), 69.9 (t, OCH_2), 55.4 (q, OCH_3), 42.9 (t, C-6), 36.6 (t, C-8), 34.7 (t, C-4), 31.0 (t, C-10), 22.3 (t, C-5), 21.0 (t, C-9), 20.9 (q, CH_3); HRMS *m/z* calcd for $C_{26}H_{30}NaO_6^+$ $[M+Na]^+$ 461.1935, found 461.1957.

General procedure C: regioselective TIPS-protection of RAL type compounds

To a solution of the RAL type compound (1 mmol) and imidazole (156 mg, 2.3 mmol) in dry CH_2Cl_2 (10 mL) was slowly added TIPS-Cl (216 mg, 1.1 mmol). The reaction mixture was stirred at room temperature for 16 h, diluted with CH_2Cl_2 (20 mL), washed with brine, dried over Na_2SO_4 and concentrated. The residue was purified by column chromatography (hexanes/EtOAc gradient elution).

2-Hydroxy-4-(triisopropylsilyloxy)benzoic acid isopropyl ester (21)

Following general procedure C, **21** was obtained as a colorless viscous oil (350 mg, 99%); R_f 0.9 (hexanes/EtOAc, 12/1); ^{1}H NMR (200 MHz, $CDCl_3$) δ 11.0 (s, 1H, 2-OH), 7.70 (d, J = 8.7 Hz, 1H, H-6), 6.44 (d, J = 2.3 Hz, 1H, H-3), 6.39 (dd, J = 8.6, 2.3 Hz, 1H, H-5), 5.24 (sept., J = 6.2 Hz, 1H, COOCH), 1.35 (d, J = 6.3 Hz, 6H, $(CH_3)_2$CH), 1.31-1.16 (m, 3H, SiCH), 1.12-1.04 (m, 18H, SiCH$(CH_3)_2$); ^{13}C NMR (50 MHz, $CDCl_3$) δ 169.6 (s), 163.5 (s), 162.5 (s), 131.2 (d), 112.1 (d), 107.5 (d), 106.6 (s), 68.6 (d), 21.9 (q), 17.8 (q), 12.9 (d); HRMS *m/z* calcd for $C_{19}H_{32}NaO_4Si^+$ $[M+Na]^+$ 375.1962, found 375.1960.

14-*O*-(Triisopropylsilyl)zearalenone (22)

Following general procedure C (reduced scale), **22** was obtained starting from ZEN (**1**, 100 mg, 0.31 mmol) as a white solid (145 mg, 99%); R_f 0.8 (hexanes/EtOAc, 2/1); ^{1}H NMR (200 MHz, $CDCl_3$) δ 11.85 (s, 1H, 16-OH), 6.90 (d, J = 15.2 Hz, 1H, H-12), 6.30 (d, J = 2.5 Hz, 1H, H-13), 6.25 (d, J = 2.5 Hz, 1H, H-15), 5.51 (ddd, J = 15.3, 10.2, 3.8 Hz, 1H, H-11), 4.97-4.79 (m, 1H, H-3), 2.75 (ddd, J = 18.4, 12.2, 3.0 Hz, 1H, H_a-8), 2.55-2.41 (m, 1H, H_a-6), 2.34-1.93 (m, 5H, H_a-5, H_b-6, H_b-8, H_{ab}-10), 1.76-1.35 (m, 5H, H_{ab}-4, H_b-5, H_{ab}-9), 1.26 (d, J = 6.1 Hz, 3H, CH_3), 1.21-1.09 (m, 3H, SiCH), 1.02-0.93 (m, 18H, SiCH$(CH_3)_2$); 13C NMR (50 MHz, $CDCl_3$) δ 211.1 (s, C-7), 171.4 (s, C-1), 165.3 (s, C-16), 161.2 (s, C-14), 143.4 (s, C-12a), 133.4 (d, C-11), 132.1 (d, C-12), 113.0 (s, C-13), 106.7 (d, C-15), 104.1 (d, C-16a), 73.3 (d, C-3), 43.0 (t, C-6), 36.7 (t, C-8), 34.8 (t, C-6), 31.1 (t,

C-10), 22.3 (t, C-5), 21.0 (t, C-9), 20.9 (q, CH_3), 17.9 (q, SiCH(CH_3)$_2$), 12.7 (d, SiCH); HRMS *m/z* calcd for $C_{27}H_{42}NaO_5Si^+$ $[M+Na]^+$ 497.6294, found 497.6299.

General procedure D: Königs–Knorr glucosylation

To a solution of the glucosyl acceptor (0.5 mmol) and bromoacetoglucose **13** (617 mg, 1.5 mmol) in dry MeCN (10 mL) was added molecular sieve 3 Å (1 g) and the resulting suspension was stirred at room temperature for 1 h. After addition of Ag_2O (174 mg, 0.75 mmol), the reaction mixture was stirred in the dark for 48 h. Since TLC analysis indicated remaining glucosyl acceptor, **13** (411 mg, 1 mmol) and Ag_2O (116 mg, 0.5 mmol) were added and stirring was continued in the dark for 24 h. The reaction mixture was diluted with CH_2Cl_2, filtered through Celite and concentrated under reduced pressure. Silica gel filtration (hexane/EtOAc gradient elution) was performed to remove most the carbohydrate byproducts. Since we were not able to fully purify the crude glucosylated products, these compounds were directly subjected to deprotection within subsequent steps.

5-Hydroxy-2-(isopropoxycarbonyl)phenyl-β,D-glucoside (17)

Following general procedure D, crude **16** was obtained starting from the PMB protected ZEN mimic **15** (95 mg, 0.3 mmol). This material was dissolved in CH_2Cl_2/H_2O (10/1, 6 mL) and cooled to 0 °C. After addition of DDQ (102 mg, 0.45 mmol) the reaction mixture was gradually warmed to room temperature. TLC/MS analysis indicated full conversion after stirring for 24 h. The reaction mixture was diluted with CH_2Cl_2 (20 mL), washed with satd. aq. $NaHCO_3$, dried over Na_2SO_4 and concentrated. The crude product was dissolved in THF/H_2O (4/1, 7 mL) and KOH (168 mg, 3 mmol) was added. After stirring for 2 h at room temperature, the solution was diluted with water (15 mL) and acidified to pH 6.5 by slow addition of 0.2 M aq. HCl followed by extraction with EtOAc (4 × 15 mL). The combined organic layer was dried over Na_2SO_4 and concentrated under reduced pressure. The residue was purified by column chromatography (MPLC, RP-C18, MeCN/H_2O, gradient elution) to yield the desired product **17** as a white solid (32 mg, 30%); R_f 0.5 (CH_2Cl_2/MeOH, 5/1); ^1H NMR

(200 MHz, CD_3OD) δ 7.68 (d, *J* = 8.8 Hz, 1H, H-6), 6.80 (d, *J* = 2.3 Hz, 1H, H-3), 6.52 (dd, *J* = 8.8, 2.3 Hz, 1H, H-5), 5.16 (sept., *J* = 6.3 Hz, 1H, COOCH), 4.82 (d, *J* = 7.2 Hz, 1H, H-1'), 3.94 (dd, *J* = 11.8, 1.5 Hz, 1H, H_a-6'), 3.80-3.68 (m, 1H, H_b-6'), 3.56-3.39 (m, 4H, H-2', H-3', H-4', H-5'), 1.34 (d, *J* = 6.3 Hz, 6H, $(CH_3)_2CH$); ^{13}C NMR (50 MHz, CD_3OD) δ 167.3 (s), 164.4 (s), 161.4 (s), 134.1 (d), 113.2 (s), 111.0 (d), 105.9 (d), 104.3 (d), 78.5 (d), 77.5 (d), 74.9 (d), 71.2 (d), 69.4 (d), 62.5 (t), 22.2 (q), 22.1 (q); HRMS *m/z* calcd for $C_{16}H_{22}NaO_9^+$ $[M+Na]^+$ 381.1156, found 381.1145.

Zearalenone-16-β,D-glucoside (7)

Following general procedure D, crude protected ZEN-16-glucoside was obtained starting from **22** (47 mg, 0.1 mmol). This material was dissolved in dry THF (2 mL) and the resulting solution was cooled to −10 °C. After addition of AcOH (6 mg, 0.1 mmol) and TBAF (0.1 mL, 0.1 mmol, 1 M in THF), the reaction was stirred at −10 °C for 3 h. TLC/MS analysis indicated full conversion of the starting material to the desired product. The solvent was evaporated and the residue was dissolved in THF/H_2O (4/1, 6 mL). KOH (56 mg, 1 mmol) was added and the reaction mixture was stirred at room temperature for 2 h. The solution was diluted with water (15 mL) and acidified to pH 6.5 by slow addition of 0.2 M aq. HCl followed by extraction with EtOAc (4 × 15 mL). The combined organic layer was dried over Na_2SO_4 and concentrated under reduced pressure. The residue was purified by column chromatography (MPLC, RP-C18, MeCN/H_2O, gradient elution) to yield the desired product **7** as a white solid (16 mg, 34%, 3 steps); R_f 0.4 (CH_2Cl_2/MeOH, 6/1); ^1H and ^{13}C NMR data matched that reported[2] (for additional 2D NMR spectra see Figures S1, S2 and S3); HRMS *m/z* calcd for $C_{24}H_{32}NaO_{10}^+$ $[M+Na]^+$ 503.1888, found 503.1905.

16-*O*-((2,2,2,-Trichloroethoxy)sulfonyl)-14-*O*-(triisopropylsilyl)zearalenone (24)

To a solution of **22** (47.5 mg, 0.1 mmol) in dry CH_2Cl_2 (2 mL) cooled to 0 °C was added 1,2-dimethylimidazole (28 mg, 0.5 mmol) and **23** (184 mg, 0.4 mmol). The reaction mixture was warmed

[2] Kovalsky Paris, M. P.; Schweiger, W.; Hametner, C.; Stückler, R.; Muehlbauer, G. J.; Varga, E.; Krska, R.; Berthiller, F.; Adam, G. *J. Agric. Food Chem.* **2014**, *62*, 1181-1189.

to room temperature and stirred overnight. After dilution with CH_2Cl_2 (10 mL), the solution was washed with water, dried over Na_2SO_4 and concentrated. Column chromatography over silica gel (hexanes/EtOAc gradient elution) gave the desired product **24** as a colorless viscous oil (62 mg, 91%); R_f 0.4 (hexanes/EtOAc, 5/1); ^{1}H NMR (200 MHz, $CDCl_3$) δ 6.96 (d, J = 2.2 Hz, 1H, H-13), 6.92 (d, J = 2.2 Hz, 1H, H-15), 6.45 (d, J = 15.6 Hz, 1H, H-12), 5.93 (ddd, J = 15.5, 9.5, 4.7 Hz, 1H, H-11), 5.42-5.25 (m, 1H, H-3), 4.92 (d, J = 11.0 Hz, 1H, CH_{2a}), 4.84 (d, J = 11.0 Hz, 1H, CH_{2b}), 2.65 (ddd, J = 17.6, 10.9, 3.2 Hz, 1H, H_a-8), 2.45-2.18 (m, 4H, H_{ab}-6, H_b-8, H_a-10), 2.15-1.93 (m, 2H, H_a-5, H_b-10), 1.80-1.51 (m, 5H, H_{ab}-4, H_b-5, H_{ab}-9), 1.37 (d, J = 6.3 Hz, 3H, CH_3), 1.31-1.18 (m, 3H, SiCH), 1.14-1.05 (m, 18H, SiCH(CH_3)$_2$); ^{13}C NMR (50 MHz, $CDCl_3$) δ 211.1 (s, C-7), 165.2 (s, C-1), 158.0 (s, C-16), 147.8 (s, C-14), 138.7 (s, C-12a), 134.8 (d, C-11), 128.4 (d, C-12), 118.6 (s, C-16a), 116.1 (d, C-13), 110.8 (d, C-15), 92.4 (s, CCl_3), 80.6 (t, OCH_2), 72.2 (d, C-3), 43.7 (t, C-6), 37.6 (t, C-8), 35.1 (t, C-4), 31.4 (t, C-10), 21.5 (t, C-5), 21.2 (t, C-9), 19.8 (q, CH_3), 17.9 (q, SiCH(CH_3)$_2$), 12.60 (d, SiCH); HRMS m/z calcd for $C_{29}H_{43}Cl_3NaO_8SSi^+$ $[M+Na]^+$ 707.1406, found 707.1433.

Zearalenone-16-sulfate, tetrabutylammonium salt (NBu_4-8)

To a solution of the **24** (55 mg, 0.08 mmol) in dry THF (1 mL), cooled to −10 °C, was added AcOH (5 mg, 0.083 mmol) and TBAF (83 µL, 0.083 mmol, 1 M in THF). The reaction mixture was stirred at −10 °C for 3 h and subsequently concentrated under reduced pressure. The residue was dissolved in dry MeOH (1 mL) and after addition of $HCOONH_4$ (44 mg, 0.7 mmol) and zinc dust (16 mg, 0.25 mmol), the reaction mixture was stirred for 16 h at room temperature. Filtration through Celite and evaporation of the solvent afforded the crude product, which was purified by column chromatography over silica gel (CH_2Cl_2/MeOH/NH_4OH = 10/4/1). **8** was obtained as tetrabutylammonium salt starting from **24** as a white solid (33 mg, 65%); R_f 0.5 (CH_2Cl_2/MeOH/NH_4OH, 20/5/1); ^{1}H NMR (400 MHz, CD_3OD) δ 7.01 (d, J = 1.9 Hz, 1H, Ar-H), 6.75 (d, J = 1.9 Hz, 1H, Ar-H), 6.39 (d, J = 15.7 Hz, 1H, H-12), 6.01 (ddd, J = 15.7, 10.0, 4.3 Hz,

1H, H-11), 5.33-5.24 (m, 1H, H-3), 3.26-3.19 (m, 8H, N^+CH_2), 2.72 (ddd, *J* = 17.1, 11.1, 3.1 Hz, 1H, H_a-8), 2.50 (ddd, *J* = 13.2, 8.5, 4.5 Hz, 1H, H_a-6), 2.37-2.21 (m, 3H, H_b-6, H_b-8, H_a-10), 2.10-1.97 (m, 2H, H_a-5, H_b-10), 1.85-1.70 (m, 2H, H_a-4, H_a-9), 1.69-1.54 (m, 11H, $N^+CH_2C\mathit{H_2}$, H_b-4, H_b-5, H_b-9), 1.47-1.35 (m, 11H, $N^+CH_2CH_2C\mathit{H_2}$, CH_3), 1.02 (t, *J* = 7.4 Hz, 12H, $N^+CH_2CH_2CH_2C\mathit{H_3}$); ^{13}C NMR (100 MHz, CD_3OD) δ 212.7 (s, C-7), 167.6 (s, C-1), 159.0 (s, C-14), 150.8 (s, C-16), 136.7 (s, C-12a), 132.7 (d, C-11), 128.7 (d, C-12), 117.8 (s, C-16a), 107.5 (d, C-13), 106.8 (d, C-15), 71.2 (d, C-3), 58.05 (q_Nt_H, $^2J_{CN}$ = 2.1 Hz, N^+CH_2, 4C), 43.4 (t, C-6), 37.1 (t, C-8), 34.8 (t, C-4), 31.0 (t, C-10), 23.4 (t, $N^+CH_2\mathit{C}H_2$), 21.2 (t, C-5), 21.0 (t, C-9), 19.3 (t, $N^+CH_2CH_2\mathit{C}H_2$), 18.9 (q, CH_3), 12.6 (q, $N^+CH_2CH_2CH_2\mathit{C}H_3$); HRMS *m/z* calcd for $C_{18}H_{21}O_8S^-$ $[M\text{-}NBu_4]^-$ 397.0963, found 397.0961.

2. NMR spectra

Figure S1: COSY spectrum of **7**

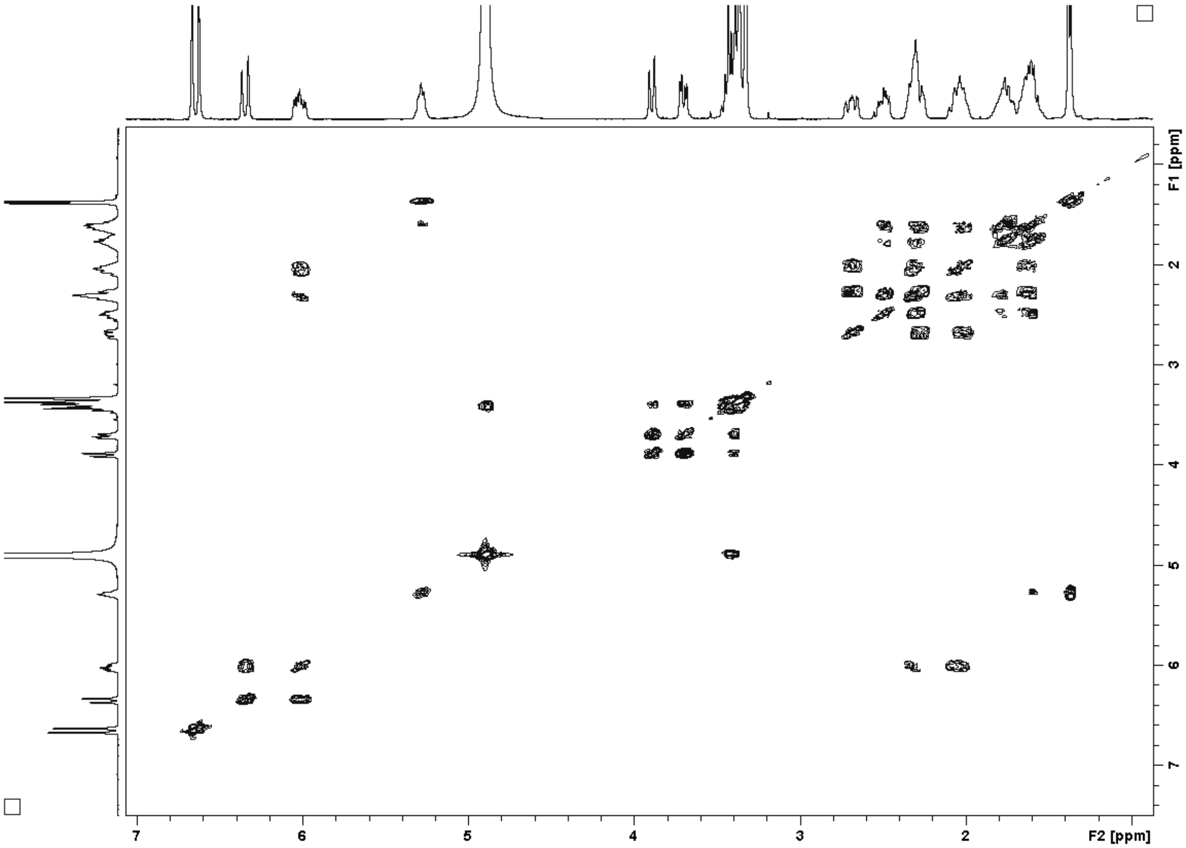

Figure S2: HSQC spectrum of **7**

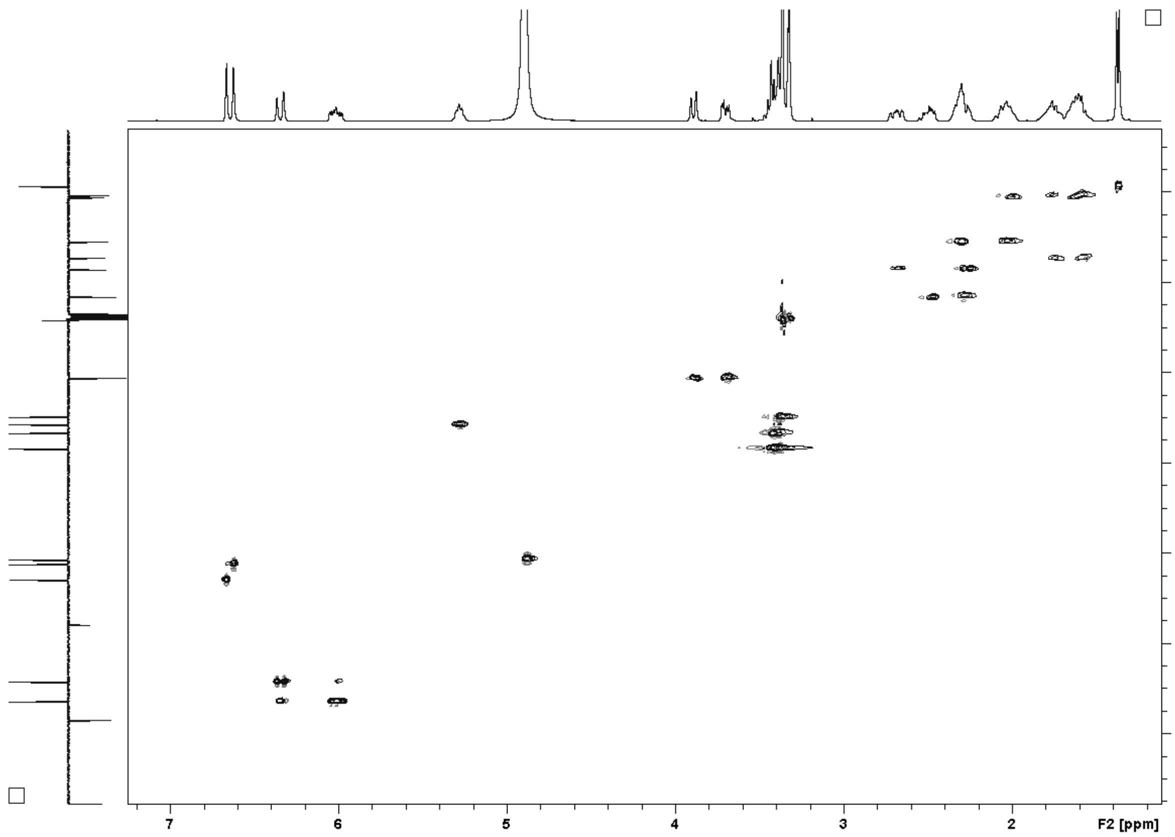

Figure S3: HMBC spectrum of **7**

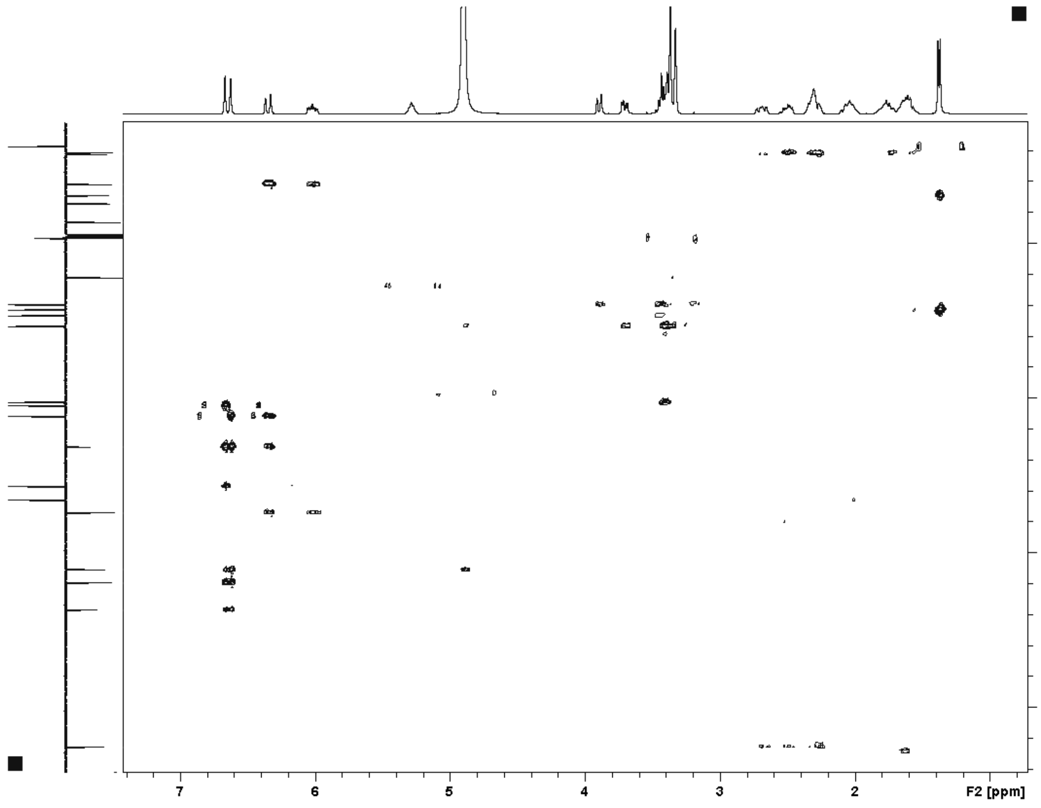

Figure S4: ^{1}H NMR spectrum of **8**

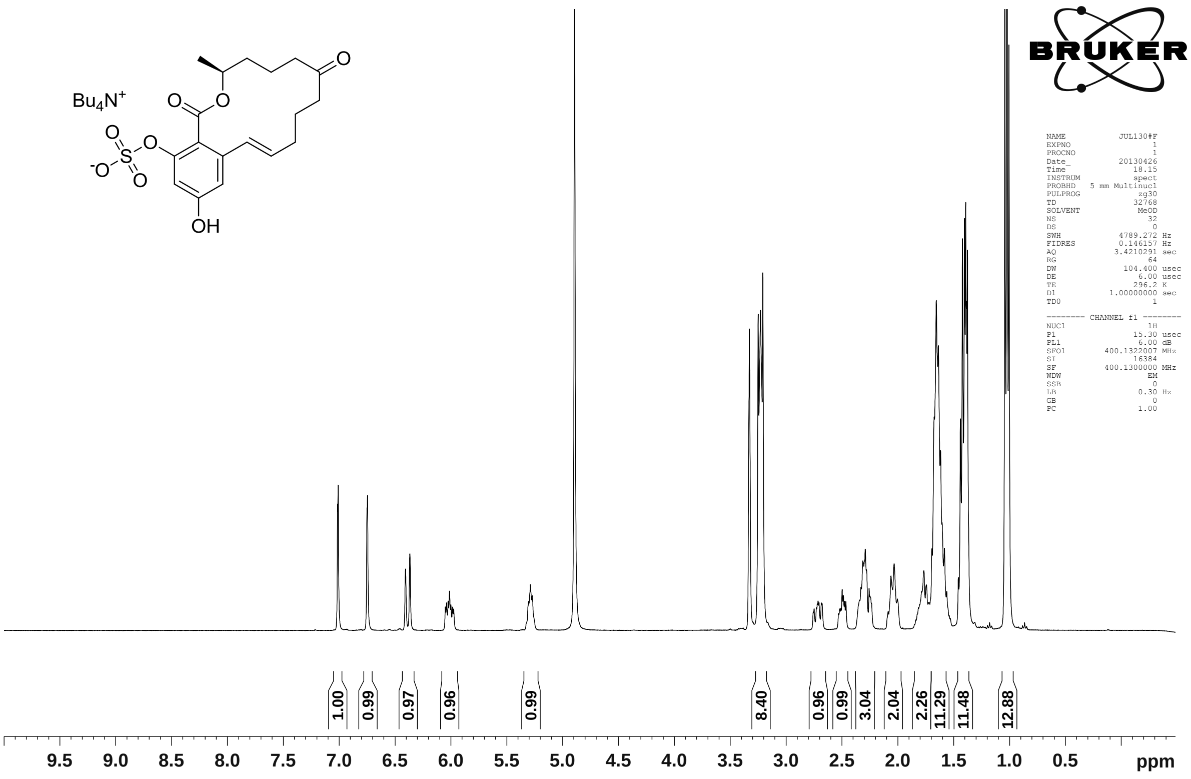

Figure S5: ^{13}C NMR spectrum of **8**

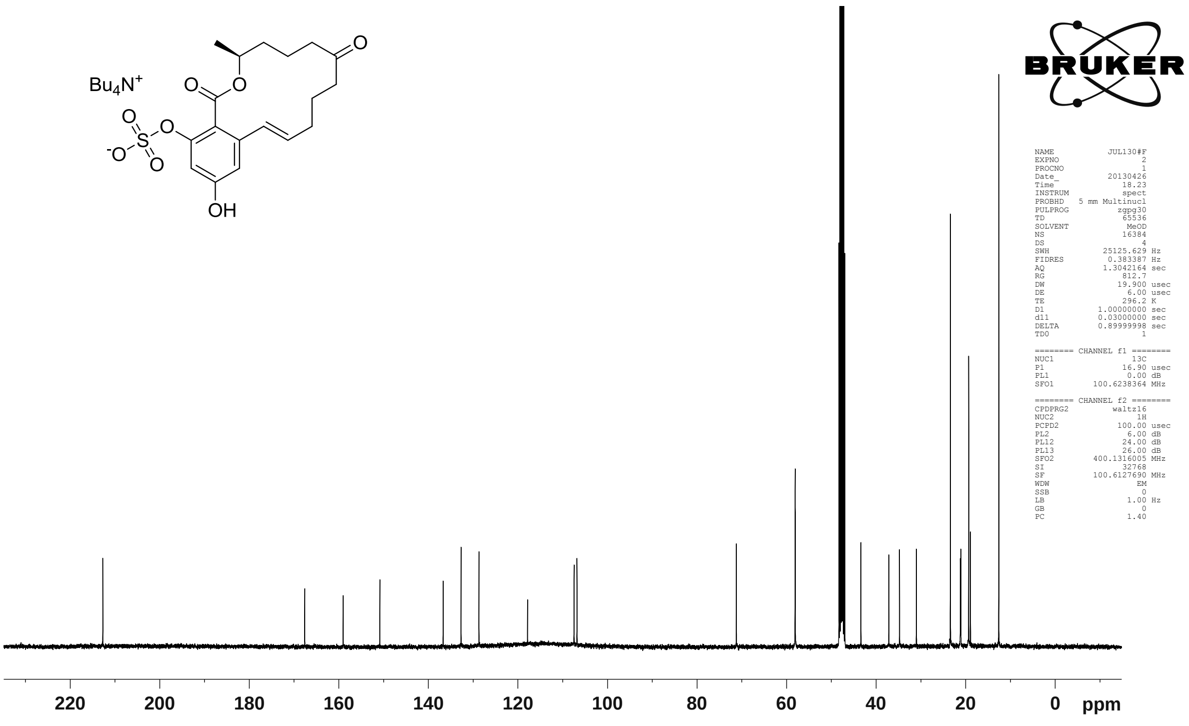

Figure S6: ^{1}H NMR spectrum of **10**

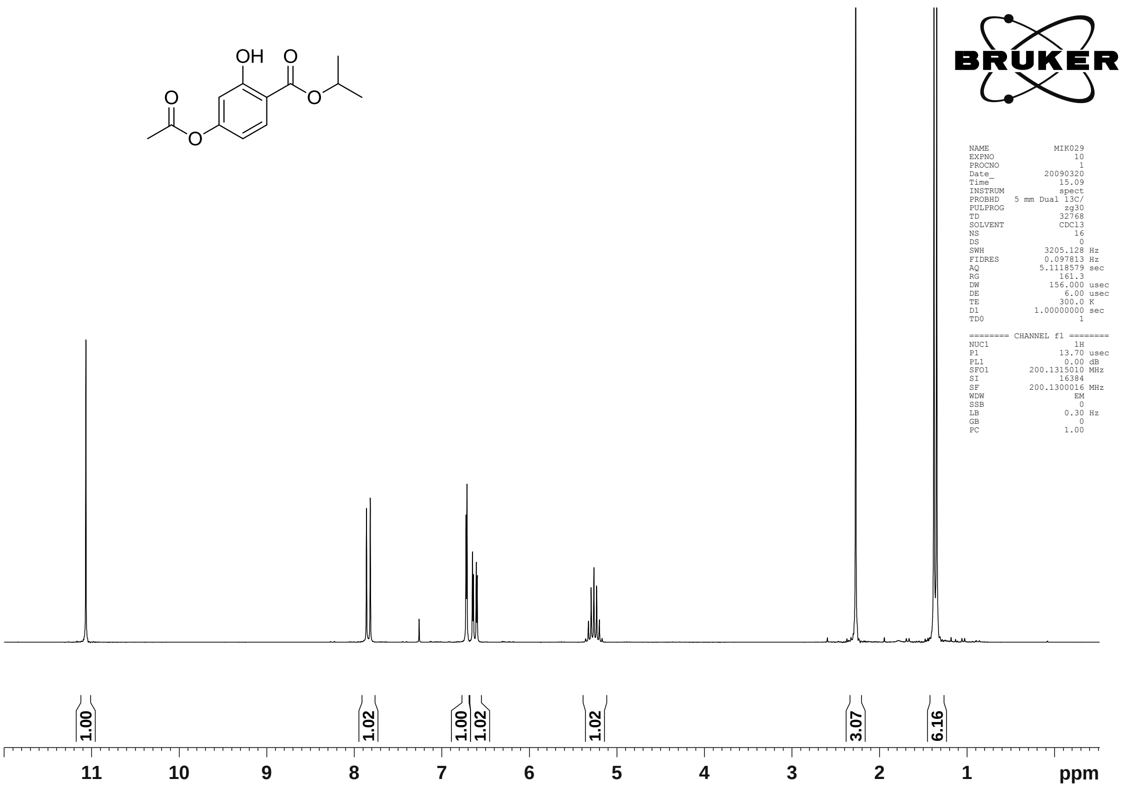

Figure S7: ^{13}C NMR spectrum of **10**

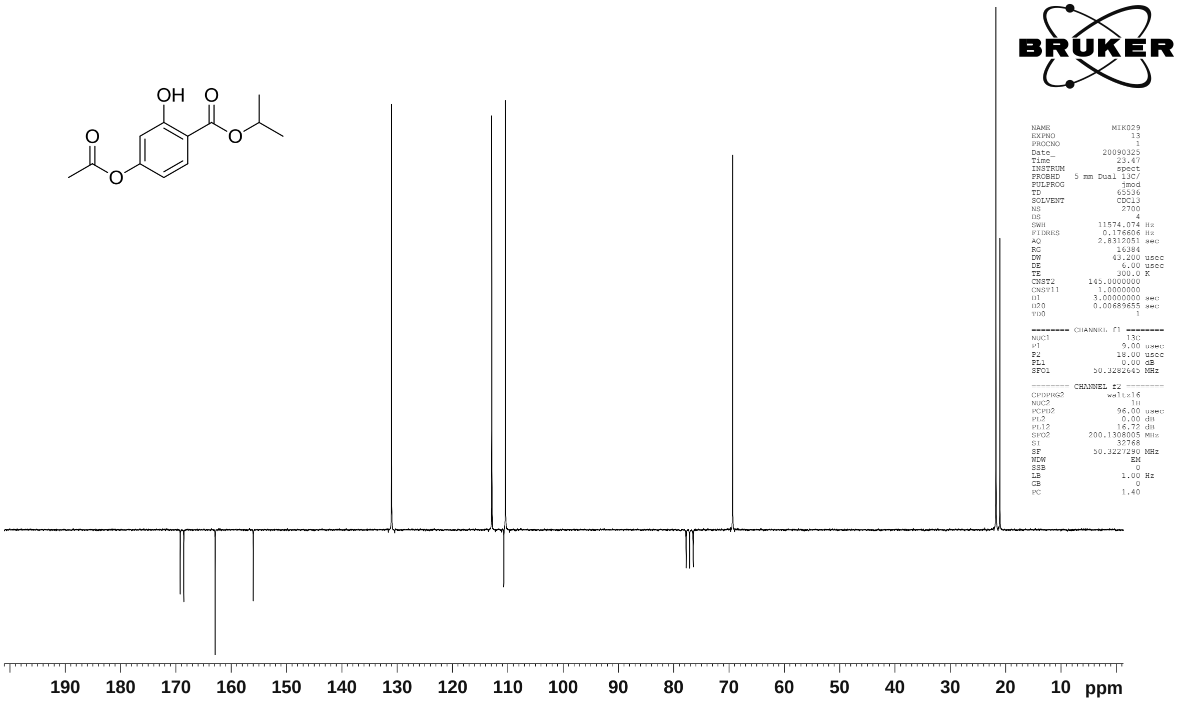

Figure S8: ^{1}H NMR spectrum of **14**

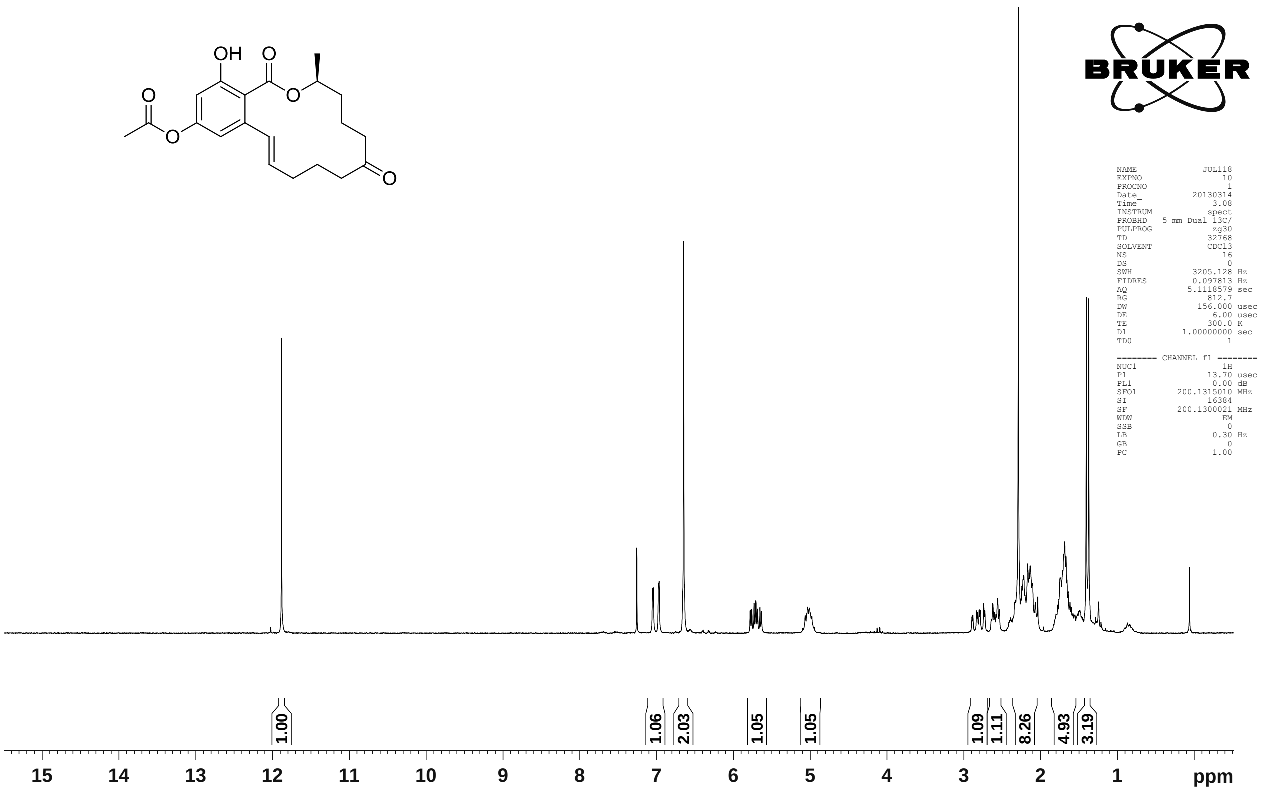

Figure S9: ^{13}C NMR spectrum of **14**

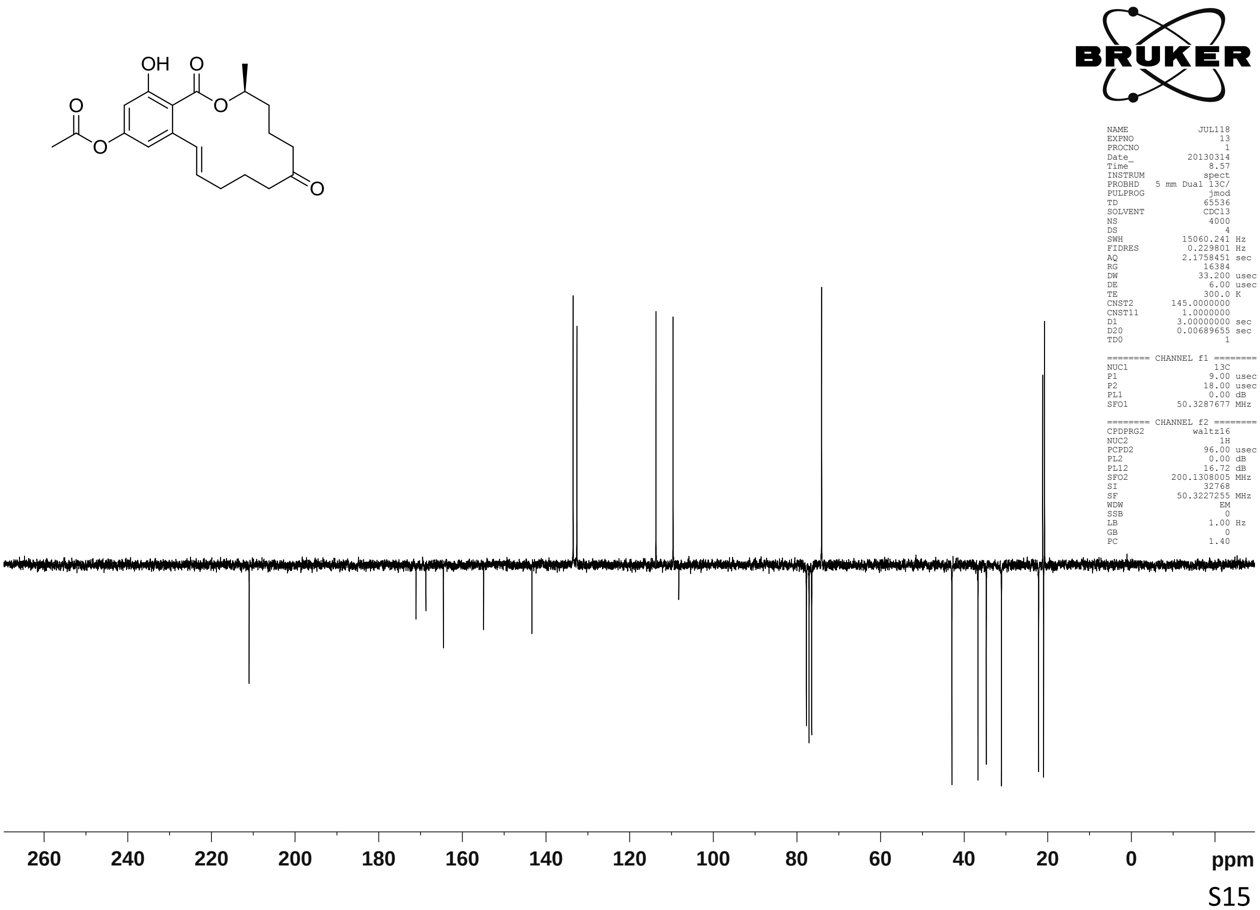

Figure S10: ^{1}H NMR spectrum of **15**

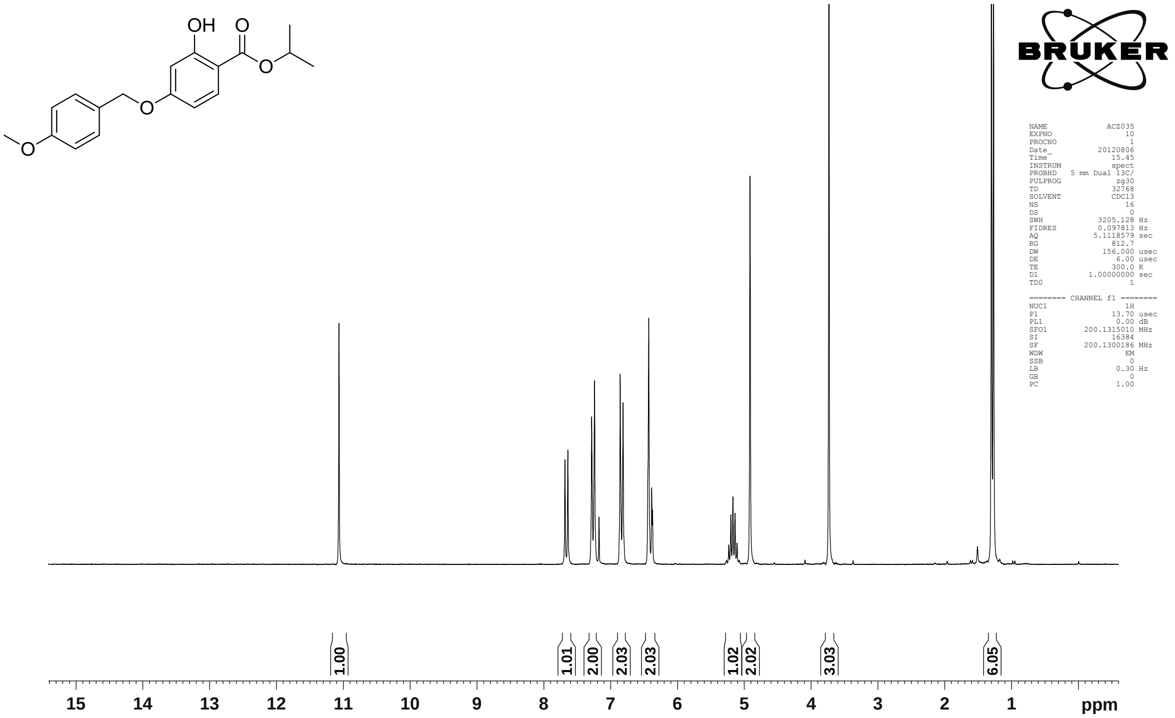

Figure S11: ^{13}C NMR spectrum of **15**

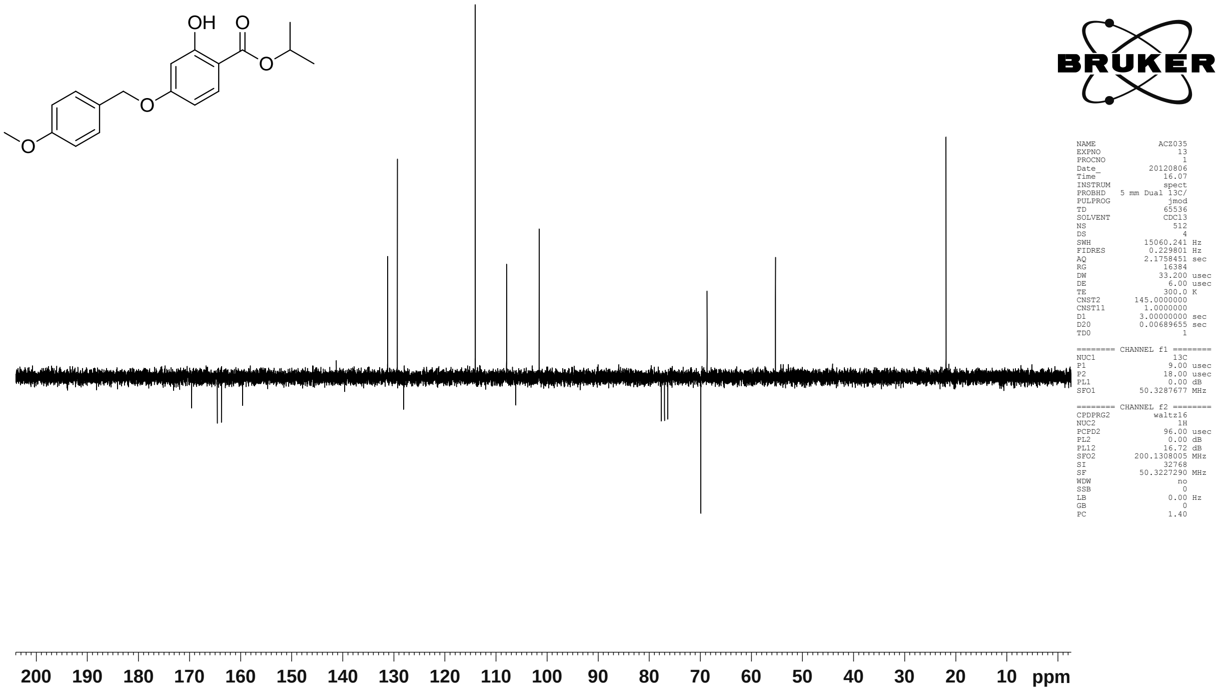

Figure S12: ^{1}H NMR spectrum of **17**

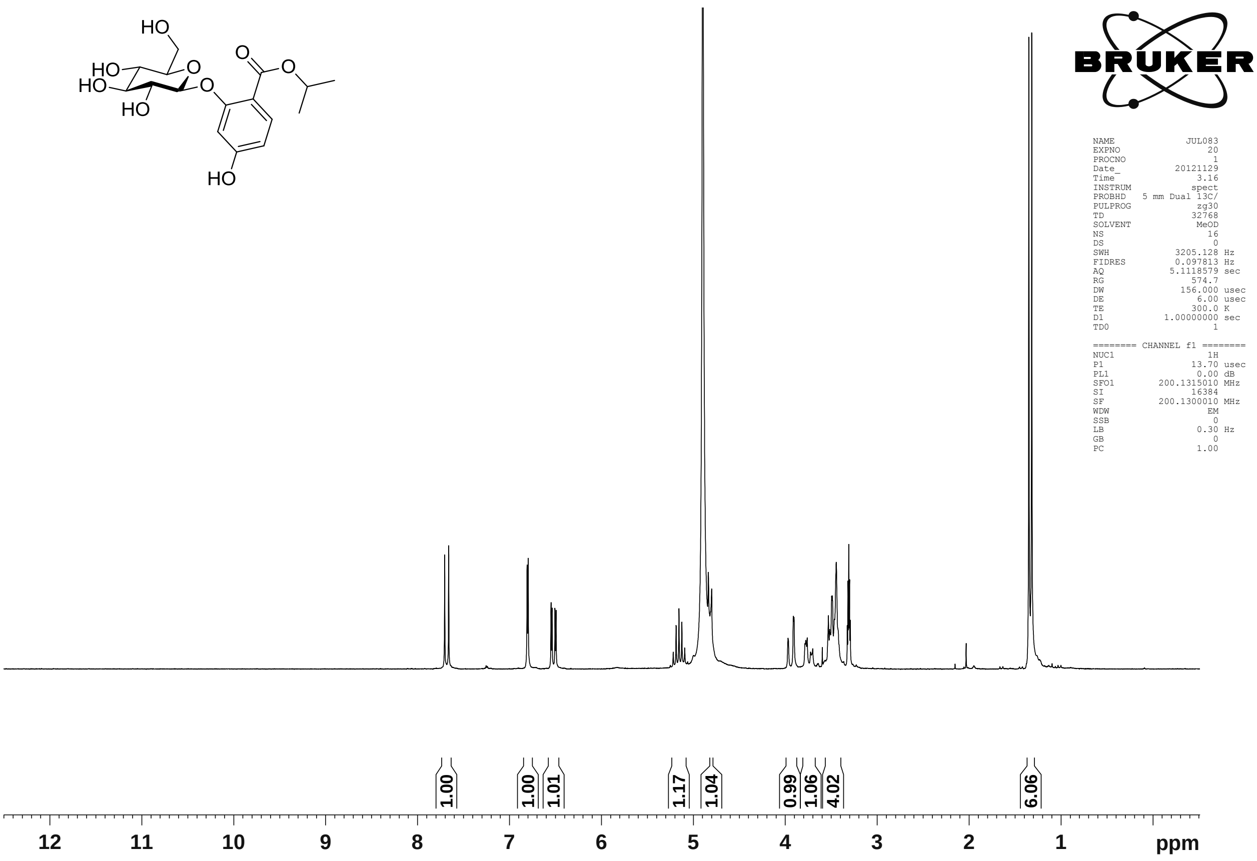

Figure S13: ^{13}C NMR spectrum of **17**

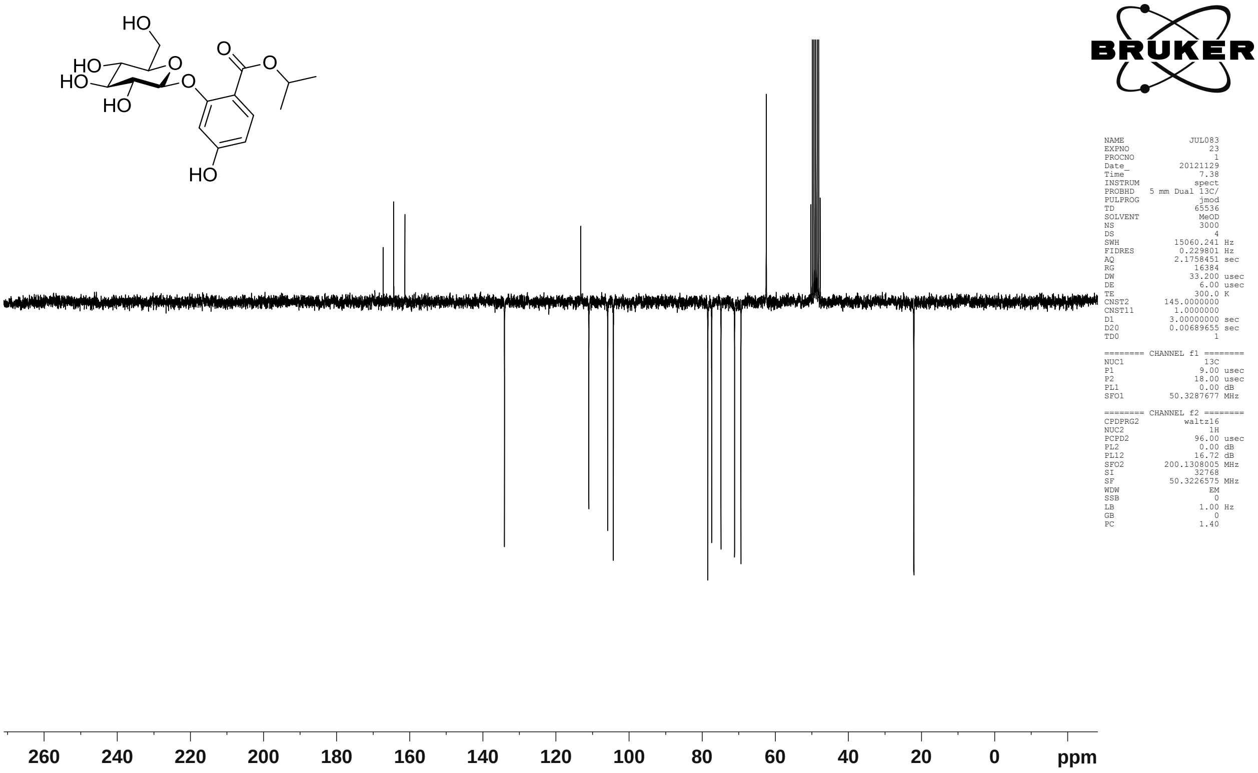

Figure S14: ^{1}H NMR spectrum **of 18**

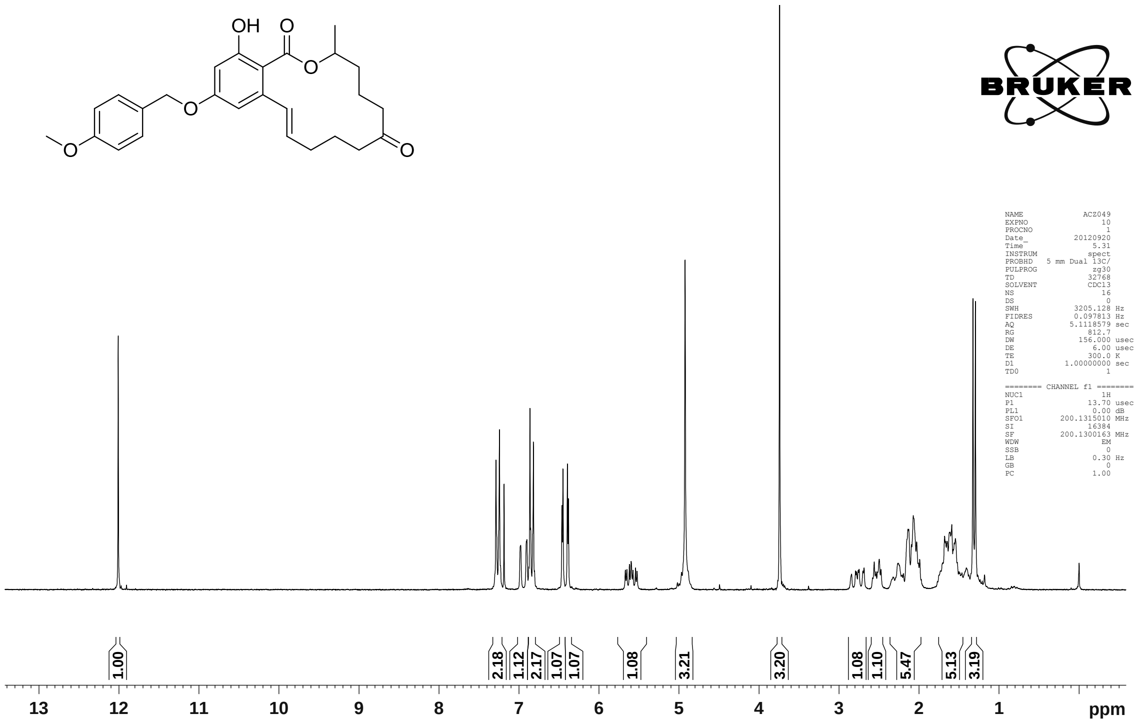

Figure S15: ^{13}C NMR spectrum of **18**

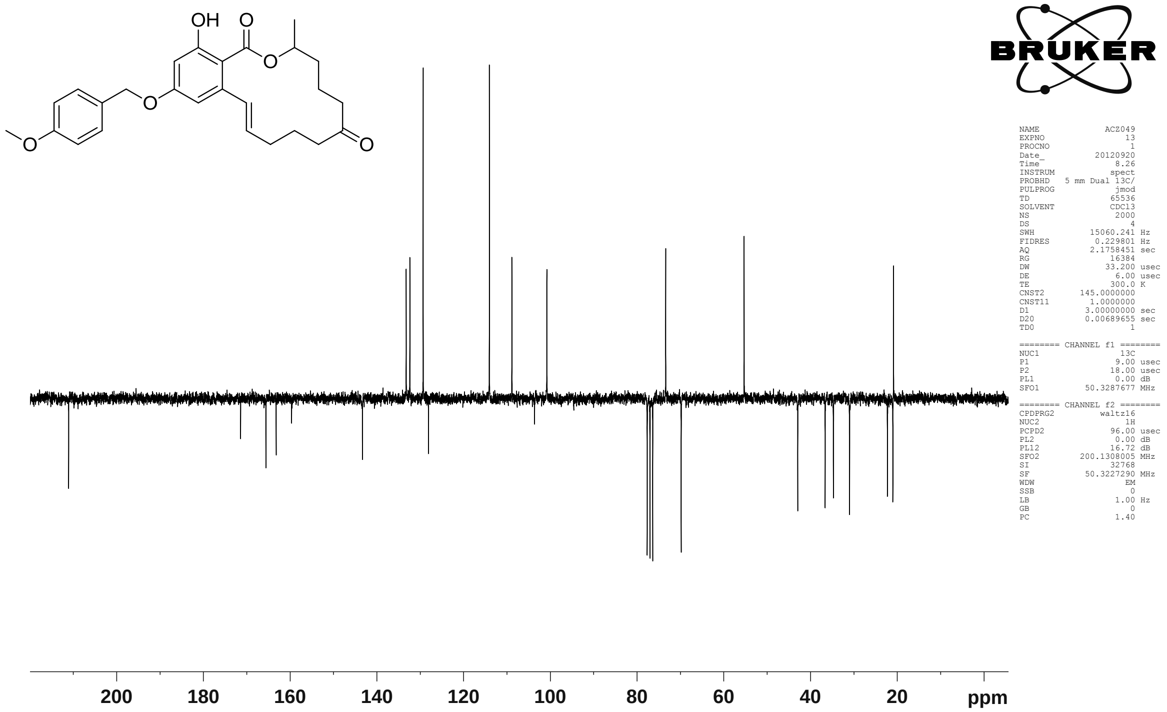

Figure S16: ^{1}H NMR spectrum of **21**

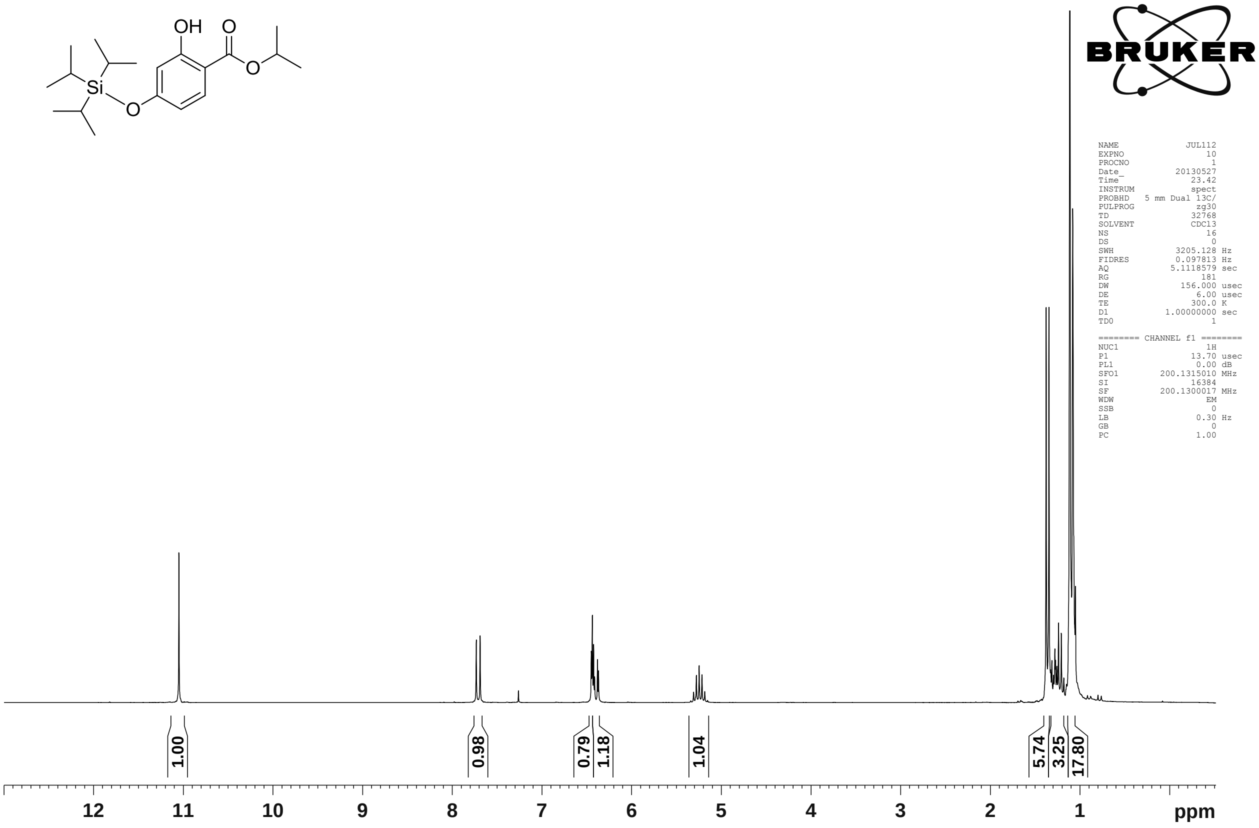

Figure S17: ^{13}C NMR spectrum of **21**

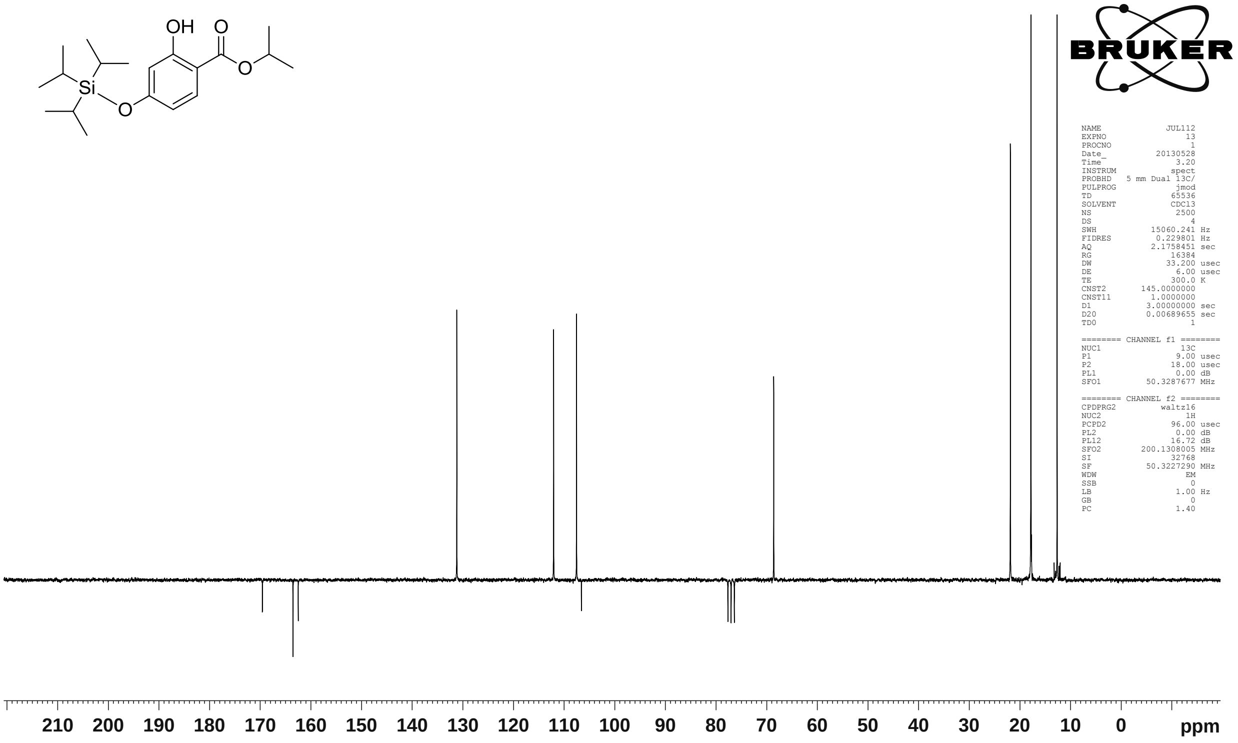

Figure S18: ^{1}H NMR spectrum of **22**

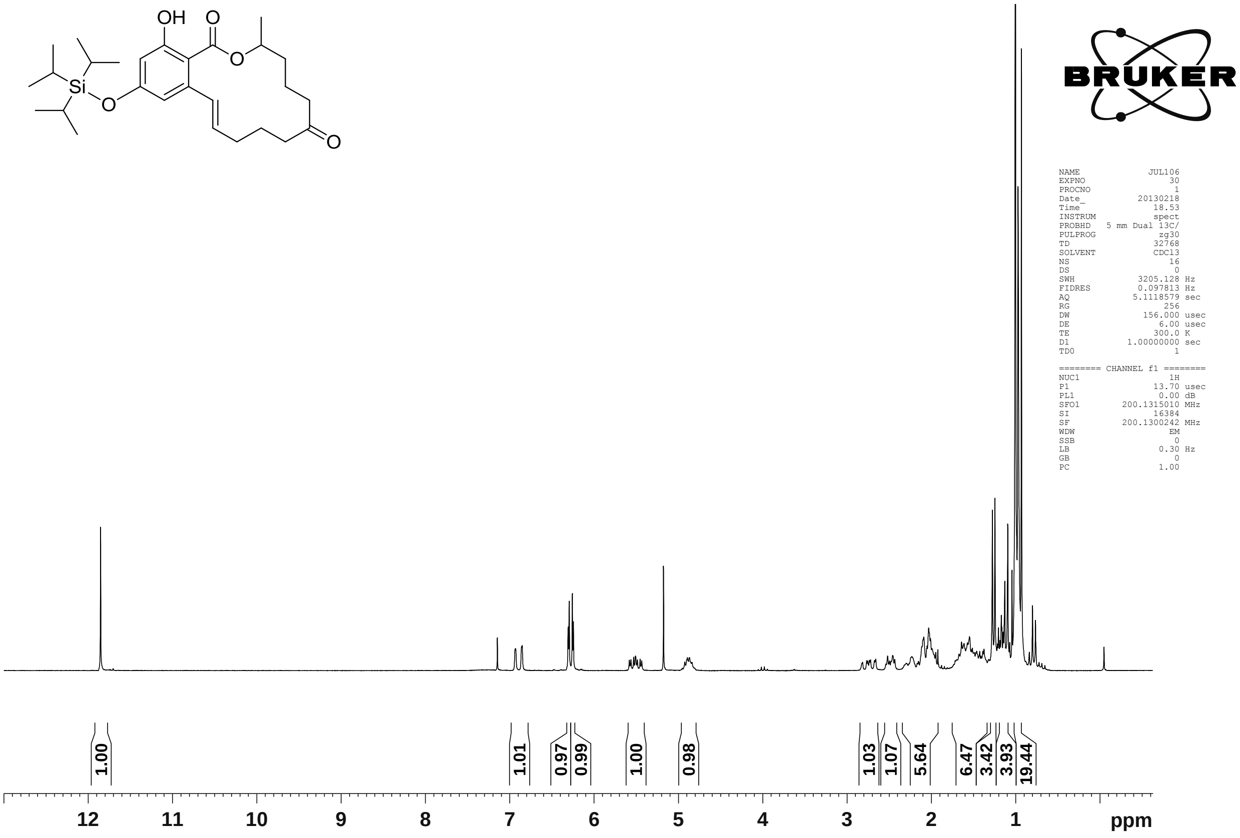

Figure S19: ^{13}C NMR spectrum of **22**

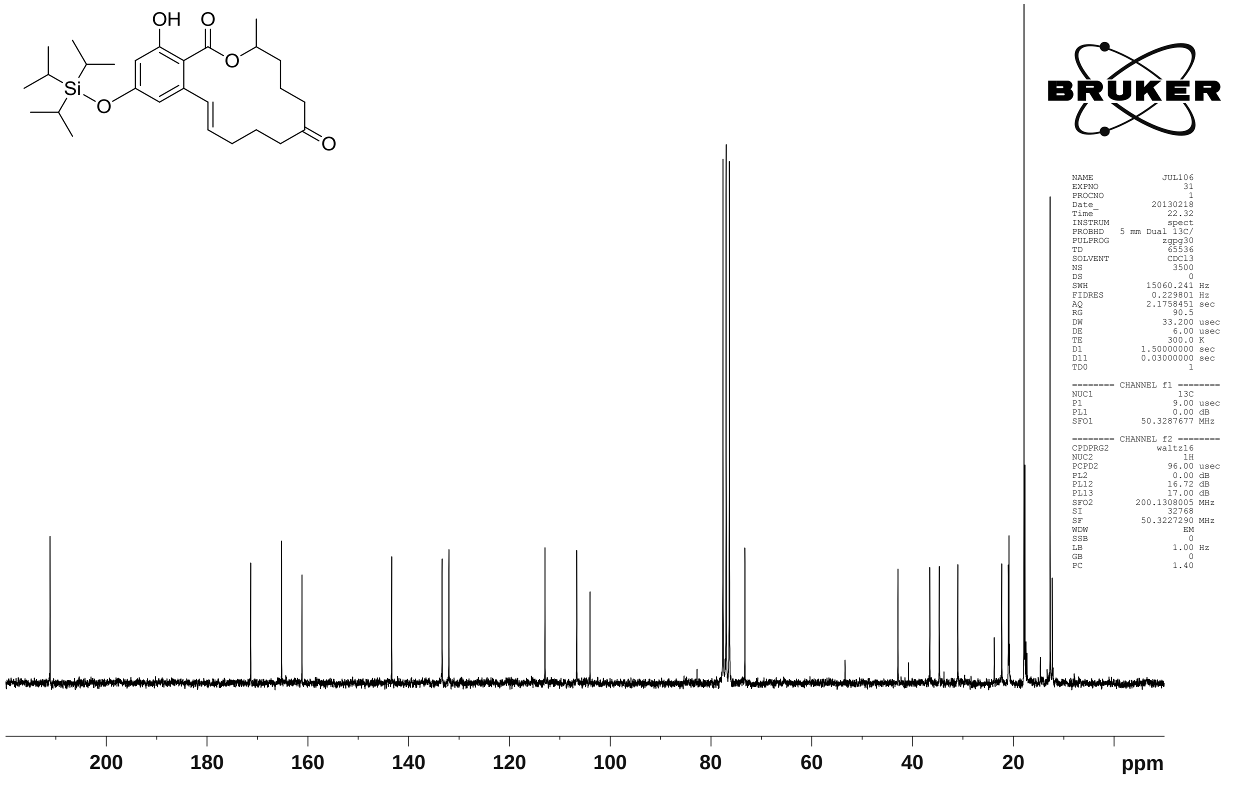

Figure S20: ^{1}H NMR spectrum of **24**

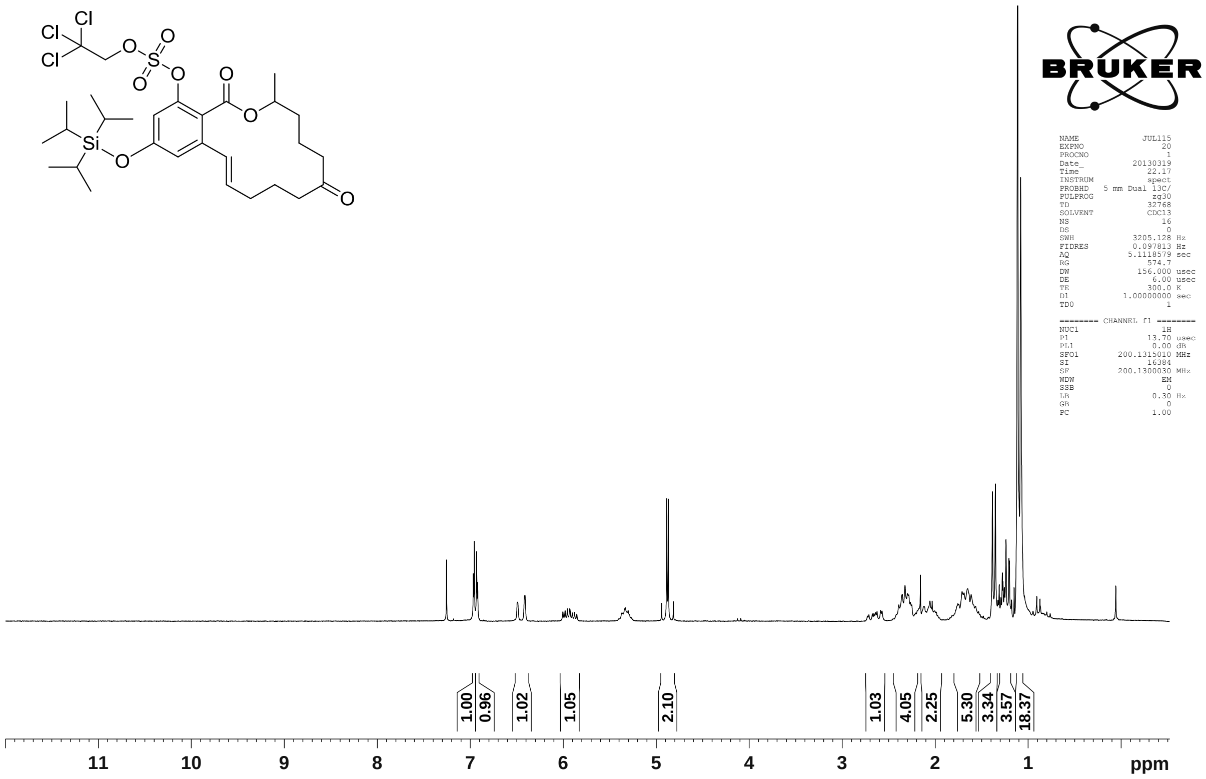

Figure S21: ^{13}C NMR spectrum of **24**

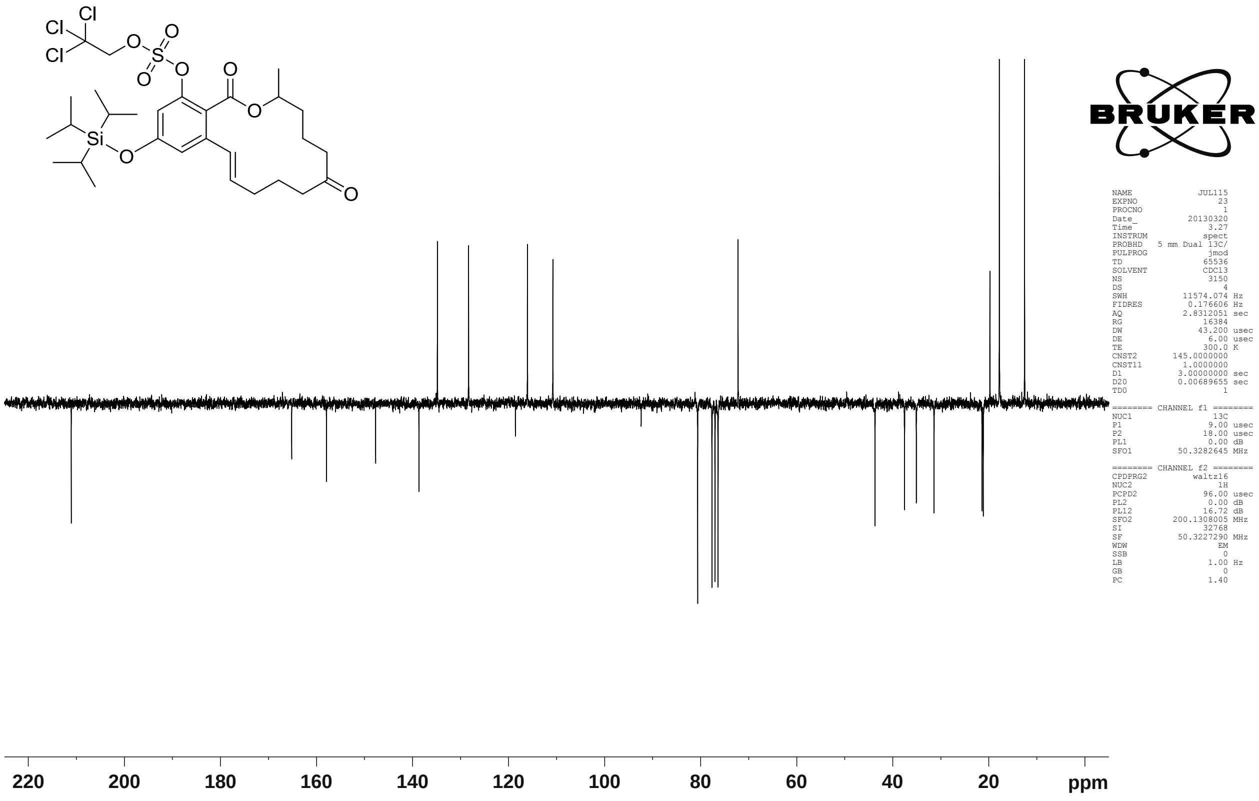

Supplement: File 1 — Experimental details (including remarks and general procedures), characterization data, copies of NMR spectra of new compounds, 2D NMR spectra of glucoside 7. [file Beilstein_J_Org_Chem-10-1129-s001.pdf]
